# RPL11 promotes non-small cell lung cancer cell proliferation by regulating endoplasmic reticulum stress and cell autophagy

**DOI:** 10.1186/s12860-023-00469-2

**Published:** 2023-03-03

**Authors:** Jie Chen, Changda Lei, Huahua Zhang, Xiaoyong Huang, Yang Yang, Junli Liu, Yuna Jia, Haiyan Shi, Yunqing Zhang, Jing Zhang, Juan Du

**Affiliations:** 1 Medical Research and Experimental Center, Medical College, Yan’an University, 716000 Yan’an, People’s Republic of China; 2 Yan’an Key Laboratory of Chronic Disease Prevention and Research, 716000 Yan’an, Shaanxi Province China; 3 Department of Gastroenterology, Ninth Hospital of Xi ’an, 710054 Xi’an, Shaanxi Province China; 4 Laboratory of Obstetrics and Gynecology, Affiliated Hospital of Yan’an University, 716000 Yan’an, Shaanxi Province China

**Keywords:** Non-small cell lung cancer (NSCLC), RPL11, Autophagy, Endoplasmic reticulum stress

## Abstract

**Background:**

Abnormal biogenesis and ribosome free function of ribosomal proteins (RPs) is important for tumorgenesis and development. Ribosomal protein L11 (RPL11) is a component of ribosomal 60 S large subunit with different roles in different cancers. Here, we aimed to unravel the role of RPL11 in non-small cell lung cancer (NSCLC), especially those affecting cell proliferation.

**Methods:**

RPL11 expression in NCI-H1650, NCI-H1299, A549 and HCC827 and normal lung bronchial epithelial cells HBE was detected using western blotting. The function of RPL11 in NSCLC cells were determined by investigating cell viablity, colony formation and cell migration. Mechanism expoloration of RPL11 effect on NSCLC cells proliferation was explored using flow cytometry, and the effect on autophagy was investigated by the additon of autophagy inhibitor chloroquine (CQ) and endoplasmic reticulum stress (ERS) inhibitor tauroursodeoxycholic acid (TUDCA).

**Results:**

RPL11 was highly expressed in NSCLC cells. Extopic expression of RPL11 promoted NCI-H1299 and A549 cells proliferation, and migration, and promoted the transition from the G1 phase to the S phase of the cell cycle. Small RNA interference of RPL11 (siRNA) suppressed NCI-H1299 and A549 cells proliferation and migration and arrested the cell cycle in G0/G1 phase. Moreover, RPL11 promoted NSCLC cell proliferation by modulating autophagy and ERS. Expression levels of autophagy and ERS markers were induced by RPL11 overexpression and inhibited by siRPL11. CQ partially suppressed RPL11-induced A549 and NCI-H1299 proliferation: CQ addition reduced RPL11-induced cells viability and clone numbers and reversed the cell cycle process. ERS inhibitor (TUDCA) partially reversed RPL11-induced autophagy.

**Conclusion:**

Taken together, RPL11 has a tumor-promoting role in NSCLC. It promotes the cell proliferation of NSCLC cells by regulating ERS and autophagy.

**Supplementary Information:**

The online version contains supplementary material available at 10.1186/s12860-023-00469-2.

## Introduction

Lung cancer is the primary cause of cancer-related mortality worldwide [1]. It can be divided into two types: small cell lung cancer (SCLC) and non-small cell lung cancer (NSCLC),of which NSCLC is the most common type, accounts for approximately 85% of primary lung cancer [2]. Unfortunately, most patients with NSCLC (47%) are diagnosed at advanced stage (stage III/IV), when the tumor has spread to multiple lymph nodes and/or distant organs; The 5-year relative survival rate for patients diagnosed with advanced stage disease is approximately 6% [3].

Ribosomal Protein L11 (RPL11) is an evolutionally highly conserved 60s ribosomal subunit [4], which is involved not only in protein synthesis but also in cell cycle progression and cell fate determination. Decreasing RPL11 expression has been reported to impede cell proliferation by reducing the ribosome content and translation capacity [5, 6]. Further, beyond its function as a ribosome componet, ribosome-free RPL11 was also reported a potential tumor suppressor that inhibited tumor cell proliferation mainly by regulating of MDM/TP53 axis and/or MYC proto-oncogene (MYC). Ubiquitin-ligase proto-oncogene (MDM2) is a negative regulator of tumor protein P53 (TP53) that destabilizes and suppresses TP53 expression by ubiquitin mediated TP53 degradation. Cytoplasmic ribosome-free RPL11, although transported from the nucleus could directly interact with and inhibit MDM2 function, thus resulting in the stabilization and activation of TP53 [7–11]. In nucleus, ribosome-free RPL11 inhibits *MYC* gene transcription directly by binding to its promoter region: by doing so, it inhibits ransformation/transcription domain associated protein recruitment and reduces histone H4 acetylation at *MYC* gene promoter region [12]. In cytoplasm, RPL11 also suppresses MYC activitiy by directly binding to and recruiting RNA-induced silencing complex to *MYC* mRNA [13, 14], interacting with MYC proteins and competing with transcriptional coactivators to suppress MYC activities [15, 16]. In contrast to the aforementioned tumor suppression function of RPL11, a previous report indicated that RPL11 could be a tumor promoter factor: increasing nuclear RPL11 expression by small nucleolar RNA SNORA18L5 promoted hepatocellular carcinoma development [17]. Therefore, RPL11 may promote tumor proliferation through mechanisms independent of the MDM2/TP53 or MYC route. However, the role and mechanism of RPL11 in the development of NSCLC remain unclear.

In this study, we aimed to explore the potential role of RPL11 and its underlying mechanisms in NSCLC. The results of RPL11 expression in NSCLC cell lines, RPL11 effects in NSCLC cells proliferation viability, migration, ERS, and autophagy using NSCLC cell lines, suggested that RPL11 promotes NSCLC cells proliferation and migration by regulating endoplasmic reticulum stress and cell autophagy.

## Materials and methods

### Cell culture and transient transfection

NCI-H1299 and NCI-H1650 cell lines were purchased from Procell Life Science and Technology Co (Wuhan, China), whereas HBE, A549, and HCC827 cell lines were gifted by the Key Laboratory of Environment and Genes Related to Diseases of Xi’an Jiaotong University. NCI-H1299, NCI-H1650, HCC827, and HBE cells were cultured in RPMI-1640 medium (Biological Industries, Beit Haemek, Israel) containing 10% fetal bovine serum (FBS, Biological Industries, Beit Haemek, Israel), A549 cells were cultured in F-12 K medium (Hyclone, Thermo Co., USA) supplemented with 10% FBS. All cells were cultured in a humidified incubator at 37 ℃ with 5% CO_2_.

For vector and small interference RNAs (siRNA) transfection, jetPRIME reagent (polyplus-transfection SA) was used according to the manufacturer’s instructions. RPL11 overexpression vector were constructed by inserting the flag-conjugated RPL11 sequence into the GV141 vector. siRNA fragments of RPL11 (siRPL11) were synthesized by GenePharma (Shanghai, China). The siRPL11 sequences are si-1: Sense, 5’-GUAUGAUGGGAUCAUCCUU-3’, Antisense, 5’-AAGGAUGAUCCCAUCAUAC-3’; si-2: Sense, 5’-GCTUAGAUACACUGUCAGAU-3’, Antisense, 5’-AUCUGACAGUGUAUCUAGC-3’; si-3: Sense, 5’-GGUGCGGGAGUAUGAGUUA-3’, Antisense, 5’-UAACUCAUACUCCCGCACC-3’. The negative control siRNA (si-nc) sequences are Sense, 5’-UUCUCCGAACGUGUCACGU-3’, Antisense, 5’-ACGUGACACGUUCGGAGAA-3’.

### Western blotting analysis

Proteins were isolated using a radio Immunoprecipitation assay buffer supplemented with a cocktail of protease and phosphatase inhibitors (TargetMol, MA, USA). Protein concentration was quantified by a BCA protein assay kit (Proteintech, Wuhan, China). Next, equivalent amounts of protein samples were separated by sodium dodecyl sulfate-polyacrylamide gel electrophoresis and transferred to a polyvinylidene difluoride membrane. For western blotting, the unbound sites of the membrane were blocked, and the membrane was sequentially incubated with primary and secondary antibodies and finally with ECL to detect the protein of interest and photographed using Syngene GBox (Syngene, Cambridge, UK). The primary and secondary antibodies used are list as follows: ribosmal protein L1 (RPL11) (Proteintech, 16277-1-AP), cyclin D1 (CCND1) (Proteintech, 26939-1-AP), heat shock protein family A (Hsp70) member 5 (HSPA5/BiP) (Proteintech, 66574-1-Ig), activating transcription factor 4 (ATF4) (Proteintech, 10835-1-AP), DNA damage inducible transcript 3 (DDIT3/CHOP) (Proteintech, 15204-1-AP), microtubule associated protein 1 light chain 3 beta (MAP1LC3B/LC3) (Proteintech, 14600-1-AP), beclin 1 (BECN1) (BOSTER, BM5181), sequestosome 1 (SQSTM1/p62) (BOSTER, M00300-1), CDK4 (Proteintech, 11026-1-AP), FLAG (Proteintech, 66008-3-1 g), phosphorylated endoplasmic reticulum to nucleus signaling 1 (phospho-ERN1/phospho-IRE1) (Affinity, AF7150), endoplasmic reticulum to nucleus signaling 1 (ERN1/IRE1) (Affinity, DF7709), actin beta (ACTB/β-actin) (Proteintech, 66009-1-Ig).

### Reverse transcriptionquantitative (RT-q) polymerase chain reaction (PCR)

Total RNA was extracted using TRIzol® reagent (Invitrogen; Thermo Fisher Scientific, Inc.), EasyScript® One-Step gDNA Removal and cDNA Synthesis SuperMix (TransGen Biotech, Beijing) were used to reverse transcribe and further examine the gene expression. The qPCR processes were as follows: 95 ˚C for 5 min; 95 ˚C for 10 s, 60 ˚C for 1 min, 72 ˚C for 30 s (40 cycles); and 94 ˚C for 90 s, 60 ˚C for 3 min gradully heating from 60 ˚C to 94 ˚C at a speed of 1 ˚C per minute. The relative gene expression levels were normalized to that of β-actin. The primer sequences were as follows:

*RPL11* Forward: 5’AAAGGTGCGGGAGTATGAGTT-3’, Reverse: 5’TCCAGGCCGTAGATACCAATG-3’ ;

*β-actin* Forward: 5’CCAACCGCGAGAAGATGA-3’, Reverse: 5’CCAGAGGCGTACAGGGATAG-3’;

*CCND1* Forward: 5’ATCTACACCGACAACTCCATC-3’, Reverse: 5’TGTTCTCCTCCGCCTCTG-3’;

*CDK4* Forward: 5’ATGGCTACCTCTCGATATGAGC-3’, Reverse: 5’CATTGGGGACTCTCACACTCT-3’.

The above experiments followed the manufacturer’s protocol. The relative expression levels were calculated using the 2^-^^∆∆Ct^ method.

### Cell counting kit-8 (CCK-8) assay

Expression vectors or siRNAs pre-transfected cells were seeded into 96-well plates and incubated for 0 h, 24 h, 48 h, and 72 h. Then the CCK-8 reagent (TargetMol, USA) was used according to the manufacturer’s instructions to determine cell viablity in proliferation .

### Clone formation assay

Pre-treated cells were seeded into 12-well plates at a density of 800 (A549) or 1000 (NCI-H1299) cells per well. After 8 days of culture, the cells were fixed using 4% paraformaldehyde, washed with phosphate-buffered saline (PBS), stained with 0.5% crystal violet(Sigma Aldrich, Merck KGaA), and imaged using a camera.

### Scratch wound healing assay

Pre-transfected cells were seeded into 6-well plates, grown to 80-90% confluency, followed by a scratch made on the monolayer in the center with a 10 µL sterile pipette tip. The cells were washed with PBS and further cultured in 1% FBS-containing medium for 72 h. Magnified images of the cells were captured at 0 h, 24 h, 48h and 72 h after wounding (Nikon, Tokyo, Japan).

### Transwell migration assay

Cell migration was examined in a 24-well cell culture transwell insert system. Transfected A549 and NCI-H1299 cells suspended in 200 µL serum-free culture medium were plated in the upper chamber and 600 µL complete culture medium were added in the lower chamber. After 48 h incubation, cells in the lower chamber were fixed with 4% paraformaldehyde for 30 min and stained with 0.1% crystal violet for 30 min. The images were captured using an inverted microscope (Nikon, Tokyo, Japan). For quantitative data, the imaged cells were further dissolved in dimethyl sulfoxide and absorbance at 570 nm was read on the SpectraMax 190 microplate reader ( MD, USA).

### Cell cycle analysis

Pre-treated cells were trypsin-digested and centrifuged; The cell pellet was collected, washed with PBS, and fixed in ice-cold 70% ethanol overnight at 4 ℃. Next, the cells were further washed with PBS and stained with 50 µg/ml propidium iodide (Biosharp, China) for 30 min. Distribution of the cell-cycle stages was examined by flow cytometry (Syngene, USA).

A**cidic vesicular organelle (AVO) staining**.

For AVO staining, 48 h after transfection, cells were further incubated in 2 µg/mL acridine orange (Med Chem Express, USA) containing medium for 20 min. The cells were then imaged using fluorescence microscopy (Nikon, Tokyo, Japan).

### Autophagy flux measurement

Autophagy flux assessment by the mRFP-GFP-LC3 method was performed to detect autophagy levels. For this, 24 h after transfection of the RPL11 overexpression vector (marked as RPL11) or its control vector, NSCLC cells were further infected with the mRFP-GFP-LC3-expressing adenovirus (Hanbio, China) for 48 h according to the manufacturer’s instruction. After that, the cells were imaged using a laser confocal microscope (Zeiss LSM800, Germany).

#### Chloroquine (CQ) and tauroursodeoxycholic acid (TUDCA) treatment

CQ (HY-17,589 A) and TUDCA (HY-19,696) were all purchased from MedChemExpress (Shanghai, China) and dissolved in DMSO. For cell treatment, CQ and TUDCA were all added 6 h after transfection at a concentration of 20 µM and 2 mM respectively.

### Statistical analysis

Experiments were all performed in three independent replicates. Data was analyzed using GraphPad Prism software version 8.0 (GraphPad Software, San Diego, USA) and presented as the mean ± SD. Unpaired student’s t-test was used to evaluate the differences in the data between two groups. One-way analysis of variance (ANOVA) was used to evaluate the differences in that data of multiple-group. *P* < 0.05 were considered to be statistically significant.

## Results

### RPL11 is highly expressed in NSCLC cells and promotes the NSCLC cells proliferation and migration

**Fig. 1:**
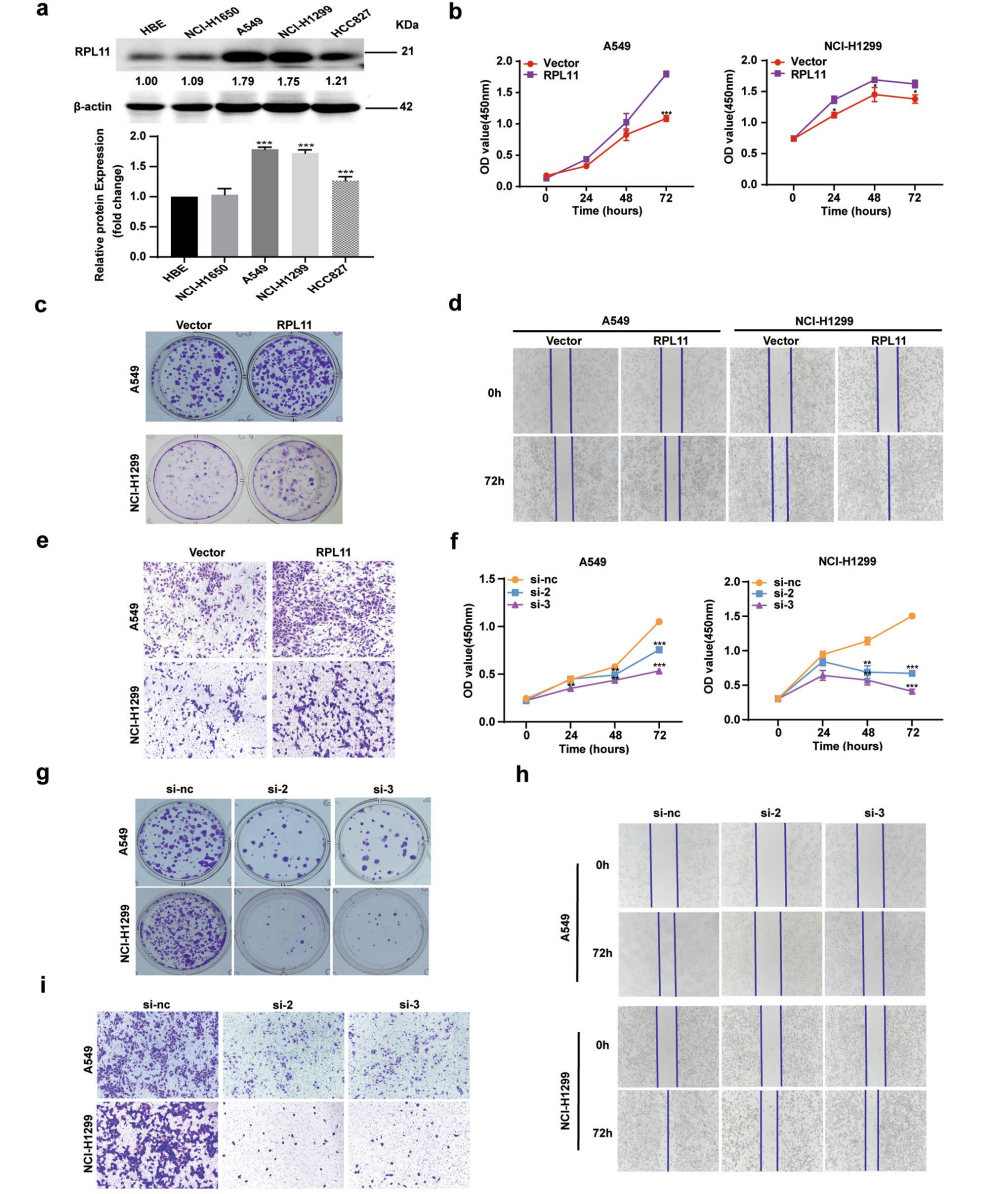
Ribosomal protein 11 (RPL11) is highly expressed in non-small cell lung cancer (NSCLC) cells and promotes cellular proliferation and migration. (a) RPL11 protein level in human bronchial epithelial HBE cells and NSCLC cell lines were detected by western blotting. (b-i) Effects of RPL11 overexpression (b, c, d and e) and siRNA knockdown (f, g, h and i) on NSCLC cell viablity (b and f), colony formation ability (c and g), and migration (d,e, h and i) were evaluated using the CCK8, colony formation, wound healing assays and transwell migration assays. Data are presented as mean ± SD from three independent trials (n = 3). Statistical data of a and f was analyzed using one-way ANOVA, statistical data of b was analyzed using unpaired student’s t-test. *P < 0.05, **P < 0.01, ***P < 0.001

To clarify the role of RPL11 in NSCLC, RPL11 expression in lung cancer cells was determined by western blotting. As Fig. 1a shows, RPL11 is relatively more highly expressed in lung cancer cells than in normal lung bronchial epithelial cells (HBE). To further explore the effect of RPL11 on the biological behavior of NSCLC cells, we overexpressed RPL11 in A549 and NCI-H1299 cells using a vector and verified its expression efficiency by western blotting and RT-qPCR (Figure S1a, b). Compared with the GV141 vector transfected control group, protein and mRNA levels of RPL11 were increased in both A549 and NCI-H1299 cells in the RPL11-overexpressed group (RPL11): these cells exhibited higher cell proliferation and colony formation capabilities than the control cells (Fig. 1b and c). Wound healing and transwell assays further confirmed RPL11 overexpression increased the migration abilities of A549 and NCI-H1299 cells (Fig. 1d and e). Meanwhile, we designed three small interfering RNA fragments of RPL11 (siRPL11), and verified their efficiency by western blotting and RT-qRCR (Fig. S1c, d): the protein and mRNA levels of RPL11 were decreased in both A549 and NCI-H1299 cells transfected with siRPL11 than in those transfected with the negative control siRNA (si-nc). Additionally, siRPL11 reduced the proliferation and colony formation abilities of A549 and NCI-H1299 cells compared with the si-nc transfected cells (Fig. 1f g). Moreover, the results of the wound healing assay showed that compared si-nc, siRPL11 transfection inhibited wound closure of A549 and NCI-H1299 cells (Fig. 1h). Transwell assay results showed that the number of A549 and NCI-H1299 cells that migrated across the chamber was significantly reduced in the siRPL11 group than in the control group, indicating an siRPL11-induced decline in cell migration (Fig. 1i). Taken together, the above results confirmed an oncogenic role of RPL11 in NSCLC by regulating cell proliferation and migration.

### RPL11 promotes cell division in NSCLC cells

**Fig. 2:**
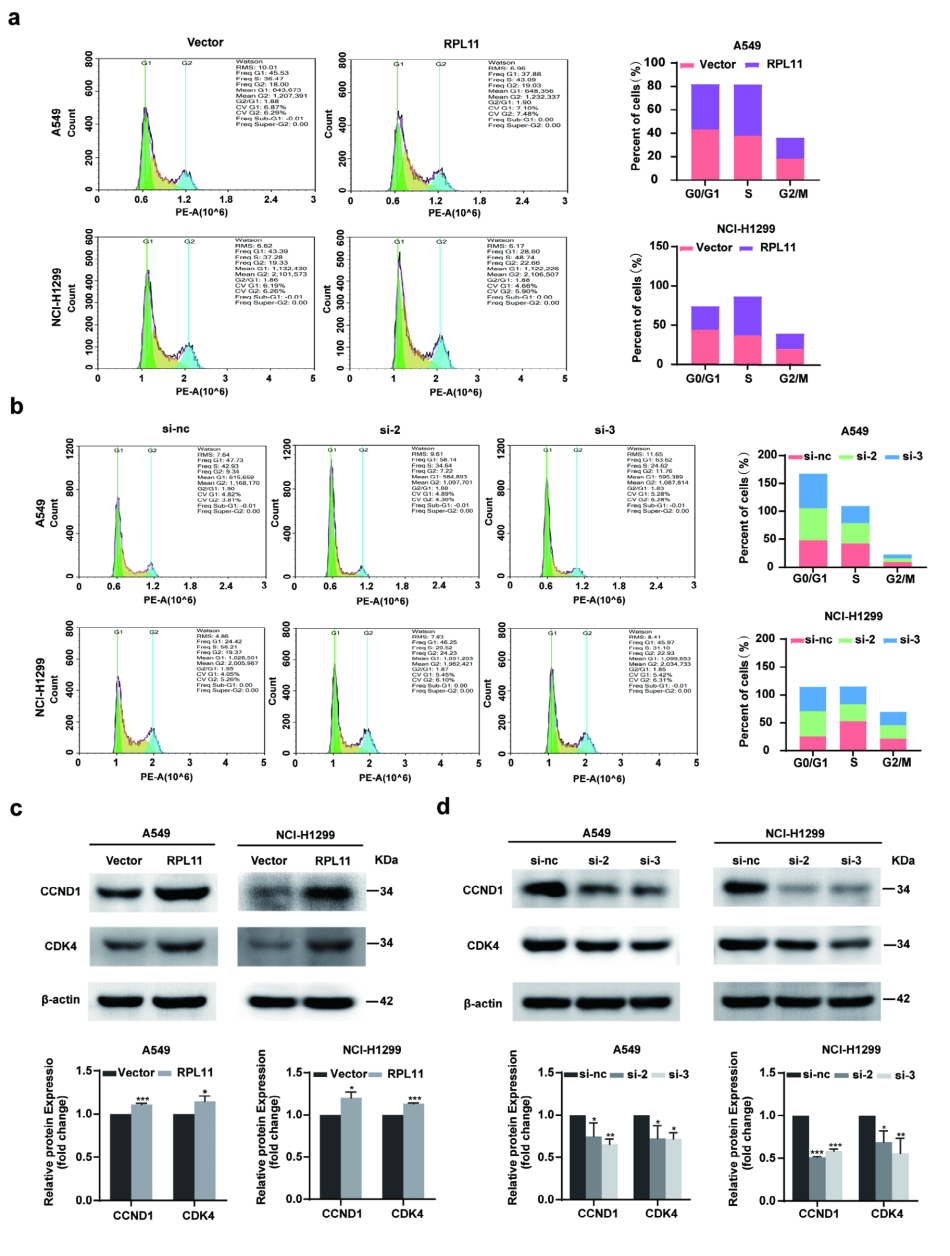
Effect of ribosomal protein L11 (RPL11) on cell cycle progression in non-small cell lung cancer (NSCLC) cells. Distribution of the cell cycle stages in NSCLC cells after overexpression (a) or siRNA-mediated knockdown (b) of RPL11 was assessed by flow cytometry. The levels of cell cycle-related proteins cyclin D1 (CCND1) and cyclin-dependent kinase (CDK4) in RPL11 overexpressed (c) or siRNA-transfected (d) groups were detected by western blotting. The blots were cut before hybridization with antibodies during blotting (c, d). The data are presented as mean ± SD from three independent trials (n = 3). Statastic data of a and c was analyzed using unpaired student’s t-test, statistic data of b and d was analyzed using one-way ANOVA. *P < 0.05, **P < 0.01, ***P < 0.001

Next, the effects of RPL11 on the distribution of cell cycle stages in NSCLC cells were examined using flow cytometry. The analysis revealed that overexpression of RPL11 promoted cell division in A549 and NCI-H1299 cells: RPL11 overexpression reduced the proportion of cells in the G0/G1 phase and increased the proportion of cells in the S phase in A549 and NCI-H1299 cells (Fig. 2a). In contrast, siRPL11 arrested A549 and NCI-H1299 cells in G0/G1 phase, as the proportion of cells in G0/G1 phase was increased and that of cells in S phase was reduced (Fig. 2b). Western blotting to determine changes in G0/G1 phase-related proteins indicated that RPL11 overexpression increased the protein levels of cyclin D1 (CCND1) and cyclin-dependent kinase 4 (CDK4) (Fig. 2c), whereas siRPL11 decreased the protein levels of CCND1 and CDK4 (Fig. 2d). Using RT-qPCR, we verified that RPL11 overexpression increased *CCND1* and *CDK4* mRNA levels, whereas siRPL11 decreased them (Fig. S1). Collectively, these data indicate that RPL11 promotes cell division of A549 and NCI-H1299 and regulates cell proliferation in NSCLC cells.

### Effects of RPL11 expression levels on NSCLC cell proliferation via autophagy

**Fig. 3:**
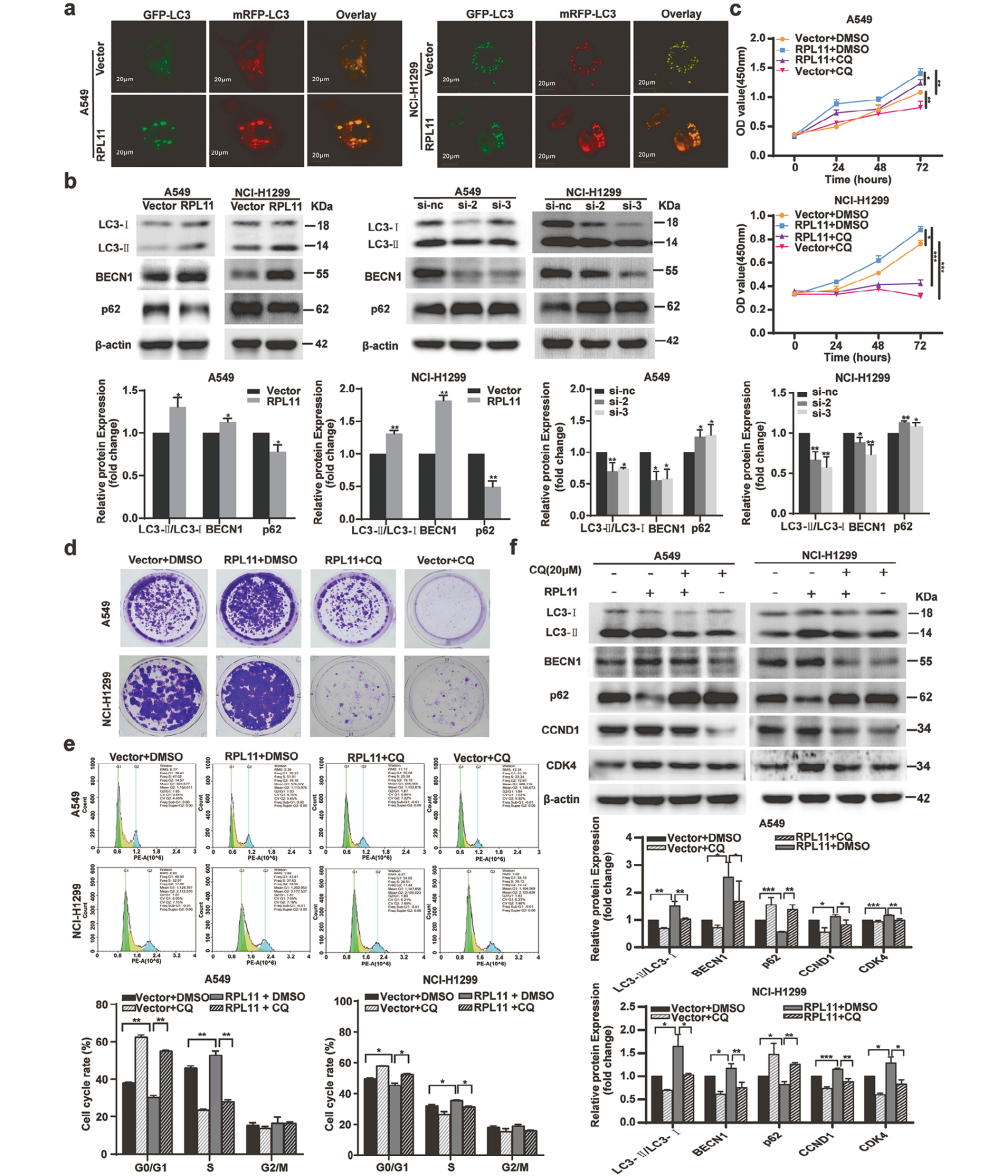
Effect of ribosomal protein L11 (RPL11) on autophagy in non-small cell lung cancer (NSCLC) cells. (a) Effect of RPL11 on autolysosome and autophagosome formation in NSCLC cells: cells transfected with RPL11 vector or its control vector GV141 (vector) were infected with mRFP-GFP-LC3 containing adenovirus: 48 h later, the cells were photographed using a laser confocal microscopy. (b) Effect of RPL11 overexpression or siRNA knockdown on autophagy-related proteins (LC3, BECN1 and p62) expression were detected by western blotting. (c-e) Effect of autophagy inhibitor chloroquine (CQ) on cell growth in RPL11-overexpressed NSCLC cells determined using the CCK-8 (**c**), clone formation (**d**), and flow cytometry (**e**) assays. (**f)** Expression levels of autophagy and G0/G1 phase-related proteins in RPL11 or its control vector transfected cells, and with the addition of 20 µM CQ or an equal volume of CQ solvent, were examined using western blotting. Blots of b and f were cut before hybridization with antibodies during blotting. The data are presented as mean ± SD from three independent trials (n = 3). Protein expression comparison between RPL11 overexpression group (RPL11) and control vector group (Vector) in b was analyzed using Student’s t-test. Statistical data of c, e, f, and comparison of proteins expression in si-nc and siRPL11 groups (b) was analyzed using one-way ANOVA. *P < 0.05, **P < 0.01, ***P < 0.001

To further explore the mechanism by which RPL11 regulates NSCLC cell proliferation, an acridine orange staining (AVO staining) that quantifies the acidic components of the cells was used. The results indicated that overexpression of RPL11 significantly promoted AVO formation in A549 and NCI-H1299 cells (Fig. S2). Thus, we speculated that RPL11 overexpression enhanced NSCLC cell atuotophagy. To confirm this speculation, an mRFP-GFP-LC3 adenovirus was used to detect intracellular autophagy flux. In this assay, a weakened GFP fluorescence indicates the fusion of lysosome with autophagosome, and red and yellow fluorescent puncta represent autolysosome and autophagosome after merging, respectively. In our study, RPL11 overexpression significantly decreased the proportion of yellow puncta while increasing the proportion of red puncta which indicated that RPL11 overexpression enhanced autophagy flux in A549 and NCI-H1299 cells (Fig. 3a). We further examined the relationship between RPL11 and autophagy-related protein expression in NSCLC cells that were transfected with RPL11 overexpression plasmids or siRPL11. The expression levels of beclin1 (BECN1) and microtubule-associated proteins 1 A/1B light chain 3B (LC3-II) increased most markedly in RPL11-overexpressed cells, whereas that of p62 was significantly decreased. Conversely, siRPL11 significantly reduced BECN1 and LC3-II expression levels, while increasing the p62 protein expression level (Fig. 3b). Moreover, chloroquine (CQ), an autophagy inhibitor blocked RPL11 overexpression-induced cell viability (Fig. 3c) and clone numbers (Fig. 3d), cell cycle progress (Fig. 3e), and reversed the autogphagy-related gene expression change (Fig. 3f).

### RPL11 promotes ERS-induced autophagy of NSCLC cells

**Fig. 4:**
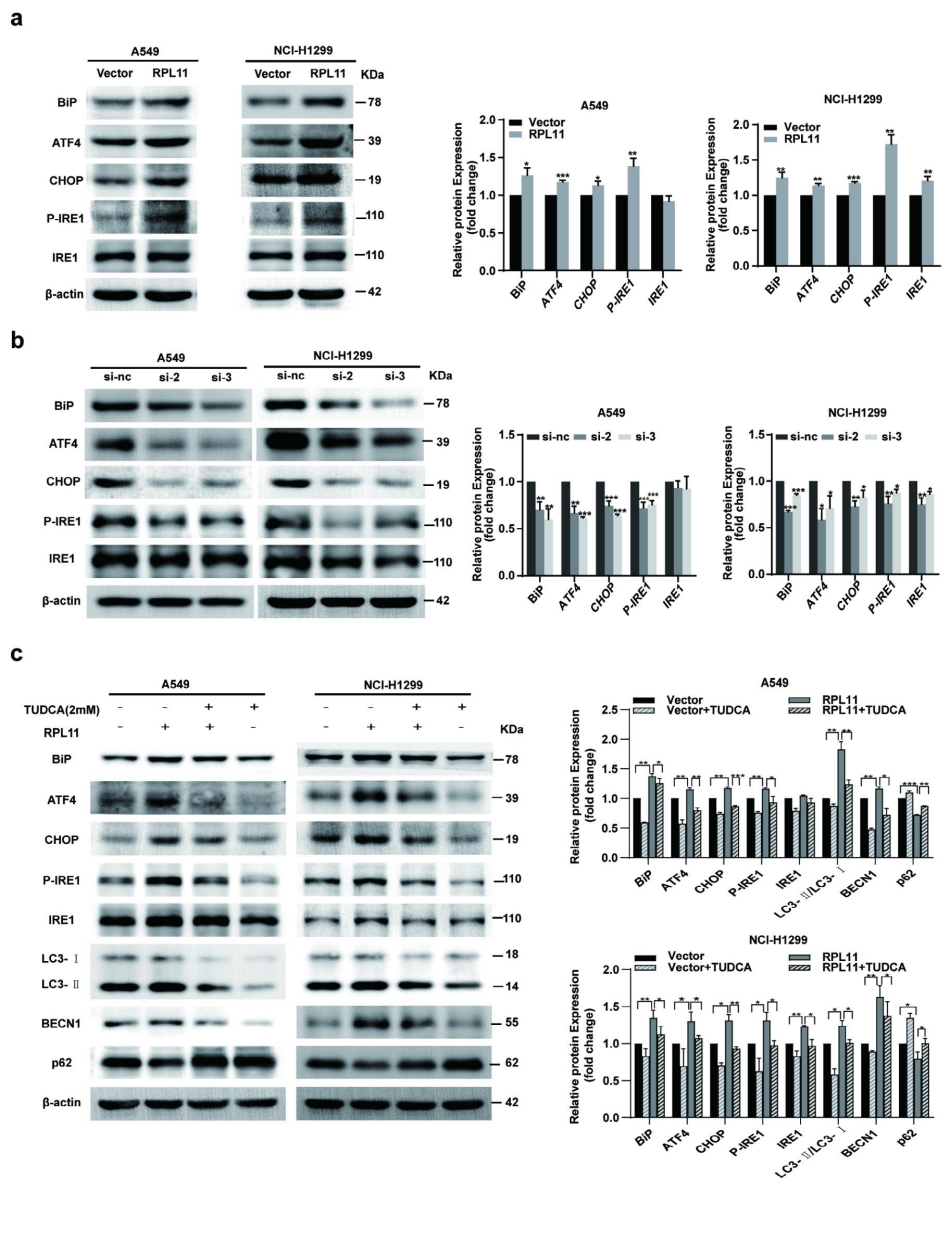
Ribosomal protein L11 (RPL11) promotes non-small cell lung cancer (NSCLC) cell proliferation through endoplasmic reticulum stress (ERS) activation. (a,b) The effect of RPL11 on ERS-related protein expression levels in NSCLC cells were examined by western blotting. (c) The effect of ERS inhibitor (TUDCA) on ERS and atuophagy-related protein expression in RPL11-overexpressing NSCLC cells was detected by western blotting. The blots (a-c) were cut before hybridization with antibodies during blotting. The data are presented as mean ± SD from three independent trials (n = 3). Statistical data of a was analyzed using Student’s t-test, statistical data of b and c was analyzed using one-way ANOVA. *P < 0.05, **P < 0.01, ***P < 0.001

Next, we explored whether ERS was involved in autophagy. Western blotting was performed to examine the expressions of ERS marker. The results showed that RPL11 overexpression in A549 and NCI-H1299 significantly increased ERS-related proteins immunoglobulin binding protein (BiP), activating transcription factor 4 (ATF4), C/EBP homologous protein (CHOP) and phospho-inositol-requiring enzyme type 1 (P-IRE1) level (Fig. 4a). Consistent with these results, siRPL11 decreased the ERS-related protein expression levels in A549 and NCI-H1299 cell compared with the control transfection group (Fig. 4b). The above data suggest that RPL11 might induce ERS in A549 and NCI-H1299 cells. Next, an ERS inhibitor, tauroursodeoxycholic acid (TUDCA), was used to block ERS. Interestingly, TUDCA not only suppressed the expression of RPL11-induced ERS-related proteins but also markedly mitigated the expression of LC3-II and BECN1 and increased the expression of p62 (Fig. 4c). Thus, RPL11 might induce autophagy in NSCLC cells by activating ERS.

## Discussion

In the present study, the role of RPL11 in NSCLC was explored. We found that RPL11 was highly expressed in NSCLC cells and it promoted the proliferation and migration of NSCLC cells. Mechanically, RPL11 could promoted NSCLC cell proliferation by regulating ERS and cell autophagy.

Ribosome biosynthesis is an energy consumption process that involves the binding of ribosomal proteins (RPs) to the ribosome RNA in the nucleolus to tightly regulate protein synthesis. In cancer development, RPs are involved in cell process of proliferation, migration, invasion, DNA damage repair, and drug resistance and thus perform tumor-promoting or suppressing activities [18, 19]. RPL11 is a previously reported cancer regulator RP, known to act as a tumor suppressor in several cancers. In breast cancer, RPL11 suppressed breast cancer cell proliferation and G1/S cell cycle transition and induced apoptosis in a TP53-MDM2-dependent way [7]. In liver cancer, RPL11-mediated MID1 interacting protein 1 depletion reduced cell viability and colony formation by inhibiting *MYC* gene expression [16]. In cervical cancer and gastric cancer, RPL11 contributed to tumorigenesis by translocating to the cytoplasm and further binding to MDM2 [20, 21]. Additionally, RPL11 cooperatively interact with ribosomal protein L5 (RPL5) to inhibited the cancer cells proliferation in the way activating TP53 or inactivating MYC [22, 23]. As RPL5 performed both tumor activator and tumor suppressor in types of cancer cells [7, 24], it’s possible that RPL11 has similar function in cancer cell growth. In lung cancer research, Xiaoping Ma et al. showed that the loss of ribosomal protein S27a expression regulated cell cycle, apoptosis, and proliferation via the RPL11-MDM2-TP53 pathway in lung adenocarcinoma cells[11]. However, they showed no direct evidence of RPL11 in regulating the lung adenocarcinoma cells proliferation. Our results presented here showed that RPL11 might be a potential tumor activator in NSCLC. First, RPL11 showed a relatively high expression level in NSCLC cell lines than in normal HBE cells, which is consistent with the results of Li H et al. who reported significantly higher levels of RPL11 proteins in lung adenocarcinoma cells tissues than in normal tissues [11]. Additionally, we investigated the functional effect of RPL11 in A549 and NCI-H1299 NSCLC cells and found that overexpression of RPL11 promoted proliferation, migration, and cell cycle progression in NSCLC cells, whereas RNA interference of RPL11 inhibited the same. As A549 is a TP53 wild-type cell line and NCI-H1299 is a TP53-null cell line, the similar effects of RPL11 overexpression or knockdown on A549 and NCI-H1299 growth indicated that, unlike other cancer types, RPL11 might regulate NSCLC cell growth in a TP53 independent way.

Cancer cells are characterized by their uncontrolled proliferation ability, unlike the precise and coordinated control of normal cell proliferation [25]. Cell cycle regulators cyclin and CDKs, which jointly regulate the cell cycle process, have proved important for cancer development in several cancers [26]. In NSCLC, overexpression of CCND1, a G1/S transition regulator, occurred in 50% of the NSCLC specimens and was considered relevant to NSCLC prognosis [27]. In this study, we found that overexpression of RPL11 increased CCND1 and CDK4 expression in A549 and NCI-H1299 cells, wherease silencing of RPL11 yielded the opposite result. These results were consistent with the flow cytometry results wherein RPL11 overexpression promoted, whereas siRPL11 suppressed the cell cycle transition from G0/G1 phase to S phase in A549 and NCI-H1299 cells, indicating that RPL11 promoted NSCLC cells proliferation partly by cell cycle control.

Autophagy is a basic cellular metabolic process that promotes cellular homeostasis, differentiation, development and survival [28]. In recent years, studies have shown that autophagy acts as a “double-edged sword” role in the development and progression of tumors [29]. However, the role of autophagy in cancer cells is relatively complex, and the inhibition or promotion of autophagy-mediated cancer may depend on the tumor’s own background or type. In this study, we first evaluated the autophagy level by acridine orange staining, and used mRFP-GFP-LC3 double fluorescence system to label and track the changes of LC3 and autophagy flux: the results showed that RPL11 overexpression promoted AVO formation and autophagic flow in A549 and NCI-H1299 cells. LC3-II, BECN1, and p62 are autophagy markers; during autophagy, LC3-I is modified and processed by the ubiquitin-like systems, including Atg7 and Atg3, to produce LC3-II with a molecular weight of 14kD. Thus, the content of LC3-II is proportional to the degree of autophagy, and the ratio of LC3-II/I estimating the level of autophagy [30]. BECN1 is an evolutionarily conserved protein and is recognized as a positive regulator of autophagy [31]. p62 is a multi-domain adaptor protein and a key player in autophagy-dependent quality control [32]. In our experimental study, western blotting detected the key autophagic molecules LC3, BECN1, and p62. RPL11 overexpression increased LC3-II and BECN1 expression and decreased p62 expression in A549 and NCI-H1299 cells; silencing RPL11 yielded the opposite result. Interestingly, we found that the autophagy inhibitor CQ reversed RPL11-induced cell proliferation and autophagy, suggesting that autophagy favoured NSCLC cell proliferation. Moreover, our previous results had shown that RPL11-induced increase in CCND1 and CDK4 expression contributed to the cell cycle transition of A549 and NCI-H1299 cells from the G0/G1 phase to the S phase. Using autophagy inhibitor CQ, we further confirmed that the RPL11-mediated NSCLC cell cycle process was autophagy-regulated. CQ reversed RPL11-induced CCND1 and CDK4 expression and the cell numbers in the S phase. Thus, our results indicated that RPL11 regulated NSCLC proliferation through an autophagy-regulated cell cycle process.

ERS is known to be closely related to autophagy. Under ER homeostasis, BiP protein binds to ERS sensors (three ER transmembrane proteins, ATF6, IRE1α, PERK) and keeps them in an inactive state. In the disease state, protein misfolding and accumulation of unfolded proteins occur. BiP proteins have a higher affinity for misfolded/unfolded proteins, and once the aforementioned ER sensors are released, they activate ERS, which, in turn, induces an unfolded protein response (UPR). The activation of UPR regulates tumor cellular processes such as cellular growth, autophagy, metastases, and angiogenesis [33]. Evidence from existing studies has highlighted the extra-ribosomal functions of RPs in UPR. RPL19 overexpression was reported to induce RES in breast cancer by pre-activation of the UPR [34]. RPL22 regulated αβ T cell development by dysregulating ERS signaling [35]. Ribosomal protein L5, an RPL11 interacting protein of 60 S ribosome unit promoted colon adenocarcinoma cell proliferation and migration, at least in part, by activating the mitogen-activiated protein kinase/extracellular signal-regulated kinase 1/2 signaling pathway [36] and also suppressed breast cancer progression by regulating ERS and autophagy [37]. Thus, targeting the UPR may be an effective strategy for treating cancer [38]. In this study, overexpression of RPL11 induced ERS. Moreover, overexpression of RPL11 enhanced LC3-II and reduced p62 protein levels, suggesting that RPL11 promotes ERS and activates autophagy in NSCLC.

## Conclusion

RPL11 was upregulated in NSCLC and promoted cellular proliferation, migration, and cycle progression in NSCLC cells. Moreover, RPL11 promoted ERS-induced autophagy of NSCLC cells. This study suggests that RPL11 might be a potential therapeutic target for NSCLC treatment.

## Electronic supplementary material

Below is the link to the electronic supplementary material.

Supplementary Material 1

Supplementary Material 2

## Data Availability

All of the data generated during this study were included in this article [and its supplementary information files].
