# Supplementary material for: RPL11 promotes non-small cell lung cancer cell proliferation by regulating endoplasmic reticulum stress and cell autophagy
Source: BMC Mol Cell Biol. 2023 Mar 3;24:7. doi: 10.1186/s12860-023-00469-2 (PMC9985270; doi:10.1186/s12860-023-00469-2)
Supplement: Supplementary file 1 — Supplementary Material 1 [file 12860_2023_469_MOESM1_ESM.pdf]

RPL11 promotes non-small cell lung cancer cell proliferation via regulating endoplasmic reticulum stress and cell autophagy

Molecular Biology Reports

Jie Chen[1,2] · Changda Lei[3] · Huahua Zhang[1,2] · Xiaoyong Huang[1,2] · Yang Yang[1,2] · Junli Liu[1,2] · Yuna Jia[1,2] · Haiyan Shi[1,2] · Yunqing Zhang[4*] · Jing Zhang[1,2,*] · Juan Du[1,2,*]

* Corresponding authors.

* Correspondence to:

Juan Du: Medical Research and Experimental Center, Medical College, Yan'an University, Yan'an 716000, People’s Republic of China

Tel: +86 13636876092; Email: dujuan123du@126.com; ORCID: 0000-0003-2410-9062

Supplementary Figures

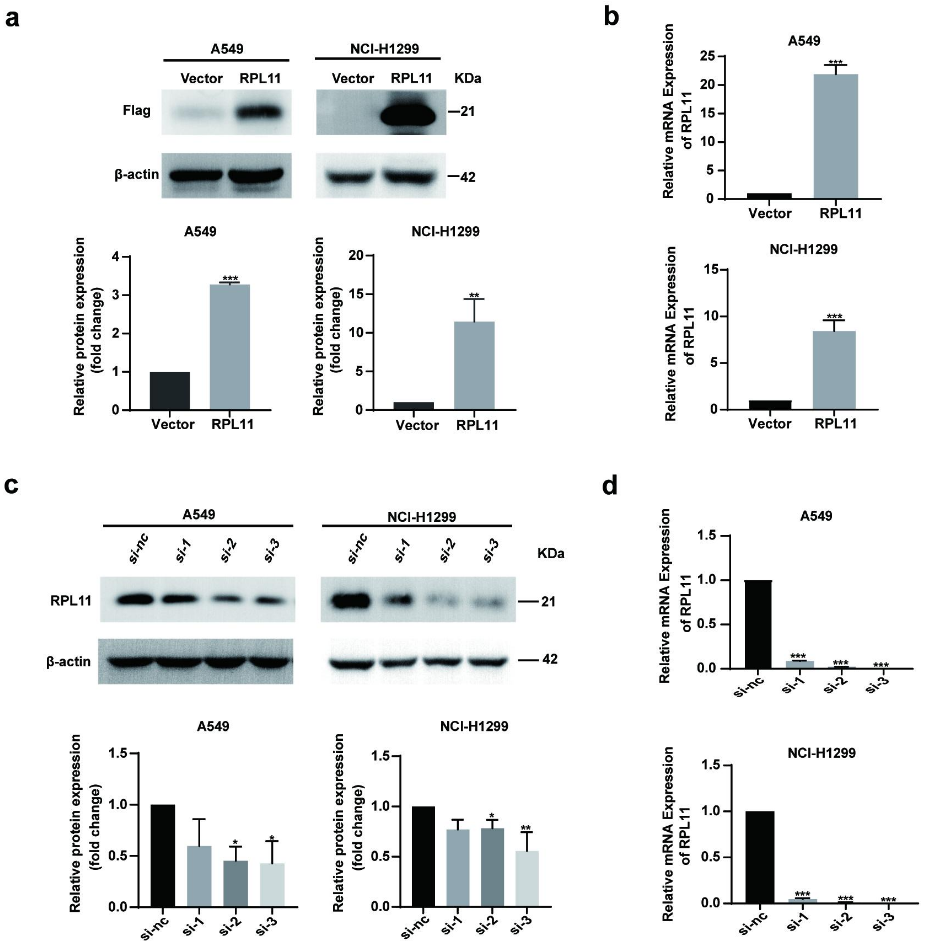

Figure S1： RPL11 overexpression vector and siRNAs（si-1,si-2 and si-3）efficiency validation. NSCLC cell lines A549 and NCI-H1299 were pretransfected with RPL11 overexpression vector (RPL11), the control vector (Vector), negative control siRNA (si-nc) or RPL11 interference RNAs (si-1, si-2 and si-3) were used to test the RPL11 expression. (a, b) Western blotting (a) and RT-qPCR (b) examination for RPL11 overexpression vector efficiency in A549 and NCI-H1299 cells. (c,d) Western blotting (c) and RT-qPCR (d) examination for siRNA knockdown efficiency of RPL11. The blots were cut prior to hybridization with antibodies during blotting. For RT-qPCR, β-actin was used as the internal control reference gene. Data are presented as mean ± SD from three independent trials (n = 3). Statistical data of a and b was analyzed using unpaired student’ s t-test, statistical data of c and d was analyzed using one-way ANOVA. *P < 0.05, **P < 0.01, ***P < 0.001

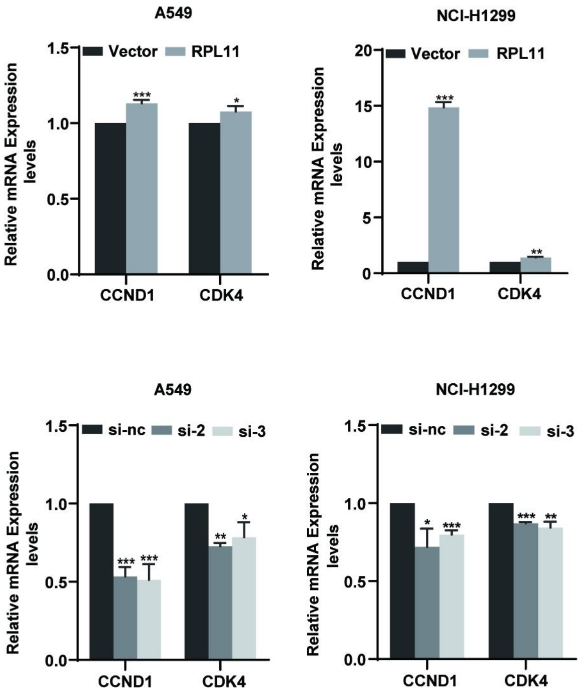

Figure S2 : Influence of RPL11 on the mRNA expression level of cell cycle-related proteins cyclin D1 *(CCND1*) and cyclin-dependent kinase (*CDK4*). A549 and NCI-H1299 cells tranfected with control vector, RPL11 overexpression vector (upper), small interference RNA of RPL11 (siRNA, si-1, si-2, si-3), or negative control RNA of RPL11 (si-nc) (lower) were RNA extracted and RT-qPCR examined for the relative mRNA expression changes of *CCND1* and *CDK4*. The relative gene expression levels were normalized to that of *β-actin.* Data are presented as mean ± SD from three independent trials (n = 3). Statistical data of the upper was analyzed using unpaired student's t-test, statistical data of the bottom was analyzed using one-way ANOVA. *$P < 0.05$, **$P < 0.01$, ***$P < 0.001$

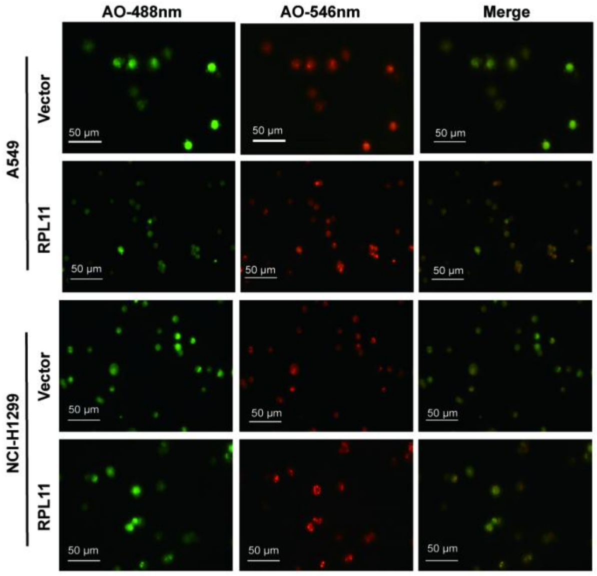

Figure S3： Acridine orange (AVO) staining for acidic components of RPL11 overexpressed NSCLC cells. A549 and NCI-H1299 cells pre-transfected with control vector (vector) or RPL11 overexpression vector (RPL11) were AVO stained and photographed. Nuclolous and cytoplasm fluoresce dim red and bright green, acidic compartments fluoresce bright red.
