# Supplementary material for: RPL11 promotes non-small cell lung cancer cell proliferation by regulating endoplasmic reticulum stress and cell autophagy
Source: BMC Mol Cell Biol. 2023 Mar 3;24:7. doi: 10.1186/s12860-023-00469-2 (PMC9985270; doi:10.1186/s12860-023-00469-2)
Supplement: Supplementary file 2 — Supplementary Material 2 [file 12860_2023_469_MOESM2_ESM.pdf]

Figure 1 a：

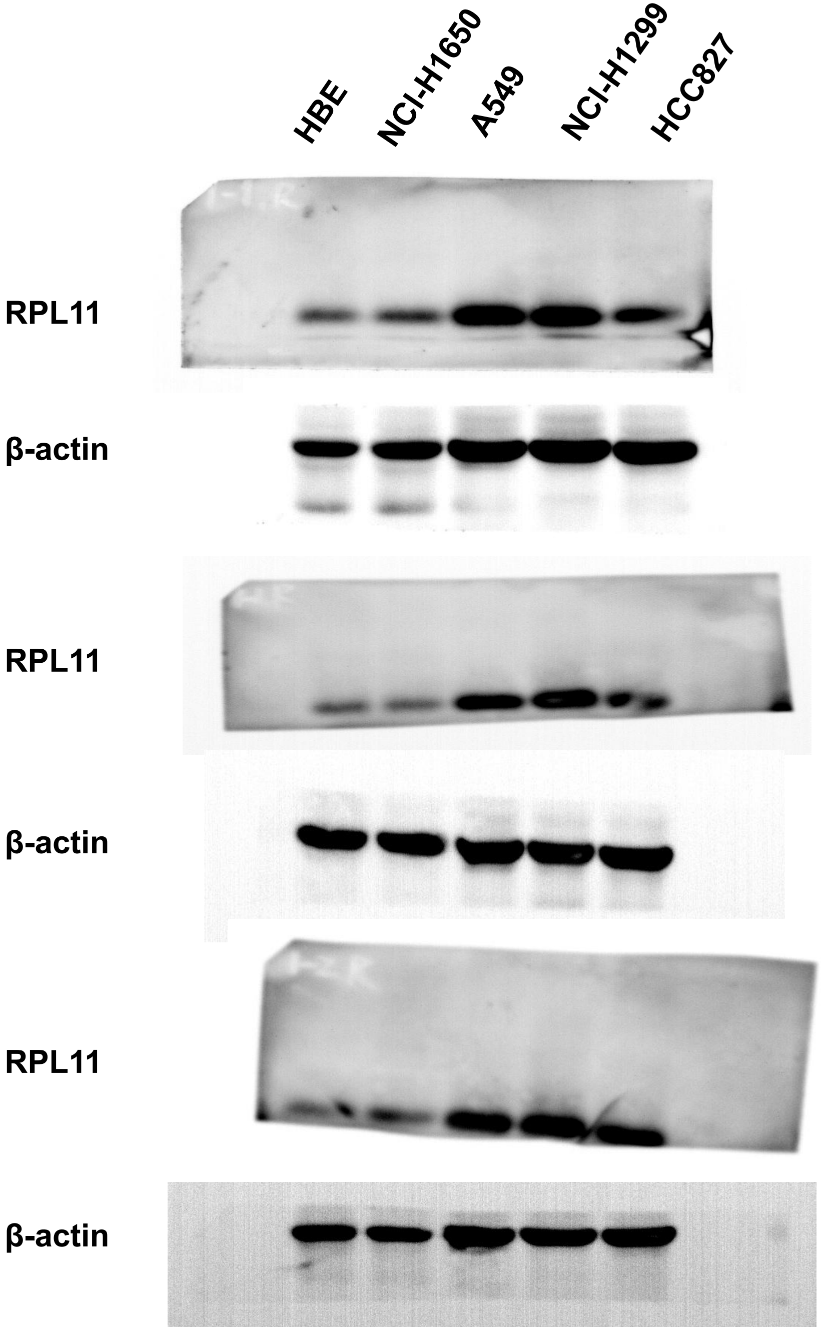

HBE
NCI-H1650
A549
NCI-H1299
HCC827
RPL11
β-actin
RPL11
β-actin
RPL11
β-actin

Figure 2 c:

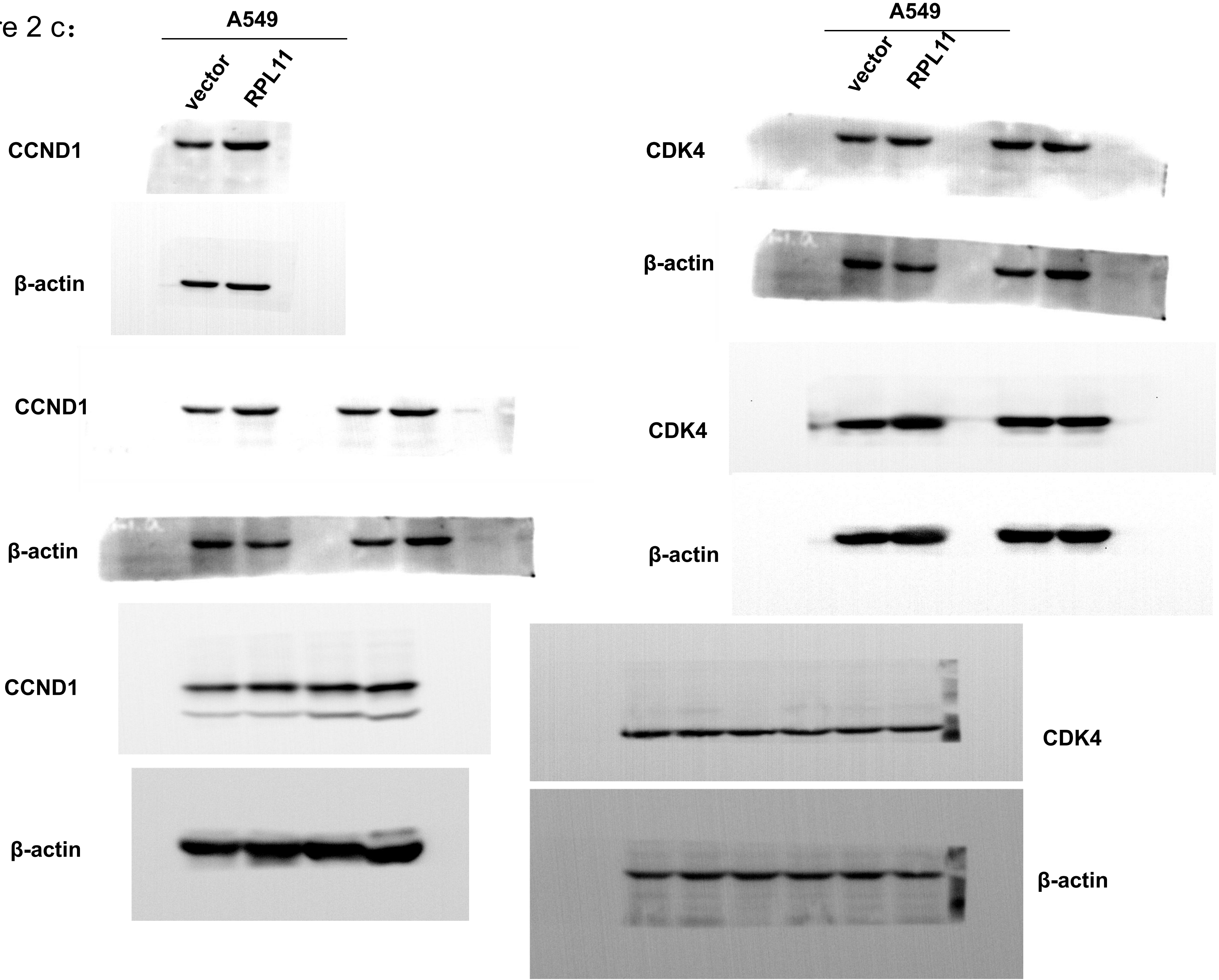
A549
vector
RPL11
CCND1
β-actin
CCND1
β-actin
CCND1
β-actin
A549
vector
RPL11
CDK4
β-actin
CDK4
β-actin
CDK4
β-actin

Figure 2 c：

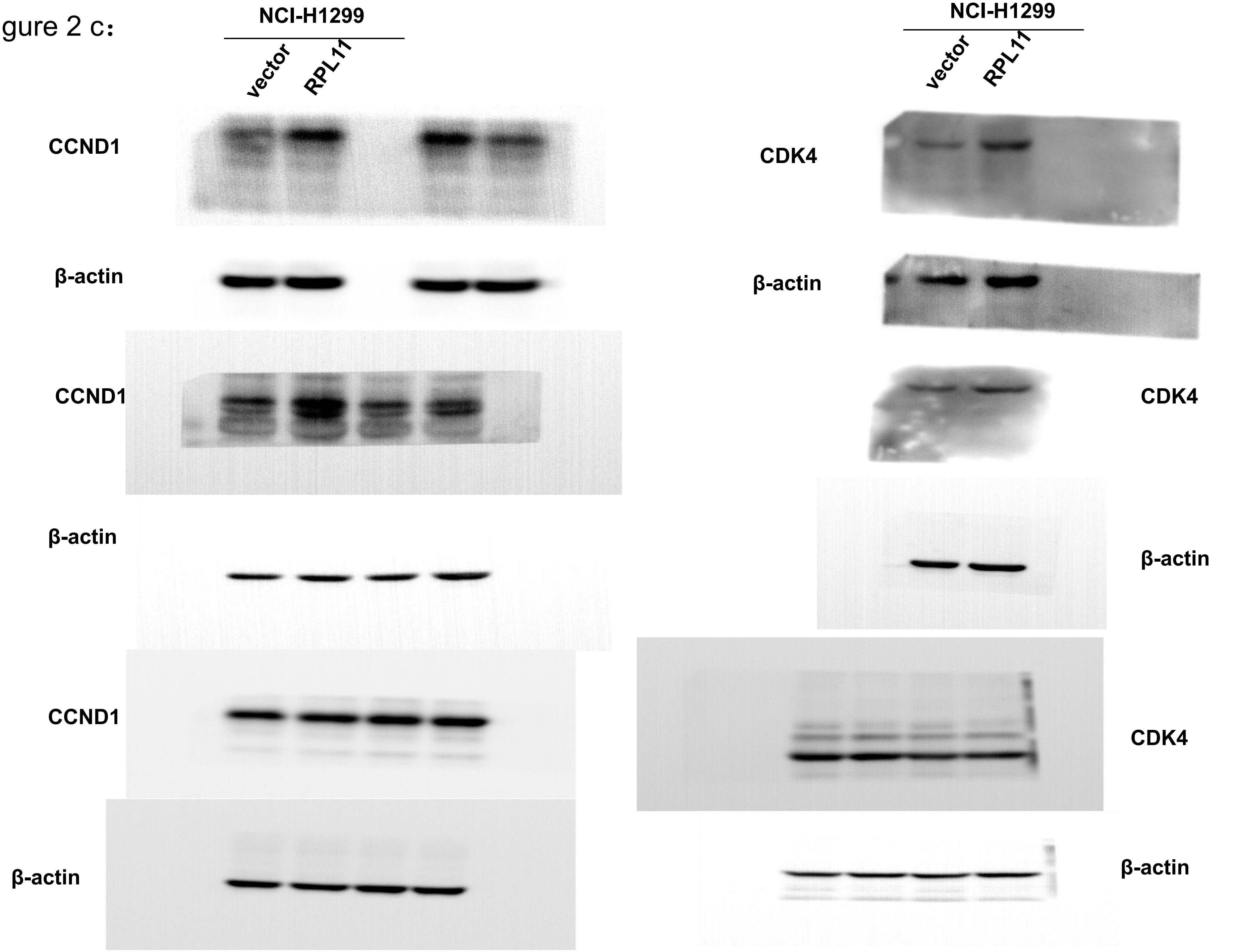
NCI-H1299
vector
RPL11
CCND1
β-actin
CCND1
β-actin
CCND1
β-actin
NCI-H1299
vector
RPL11
CDK4
β-actin
CDK4
β-actin
CDK4
β-actin

Figure 2 d：

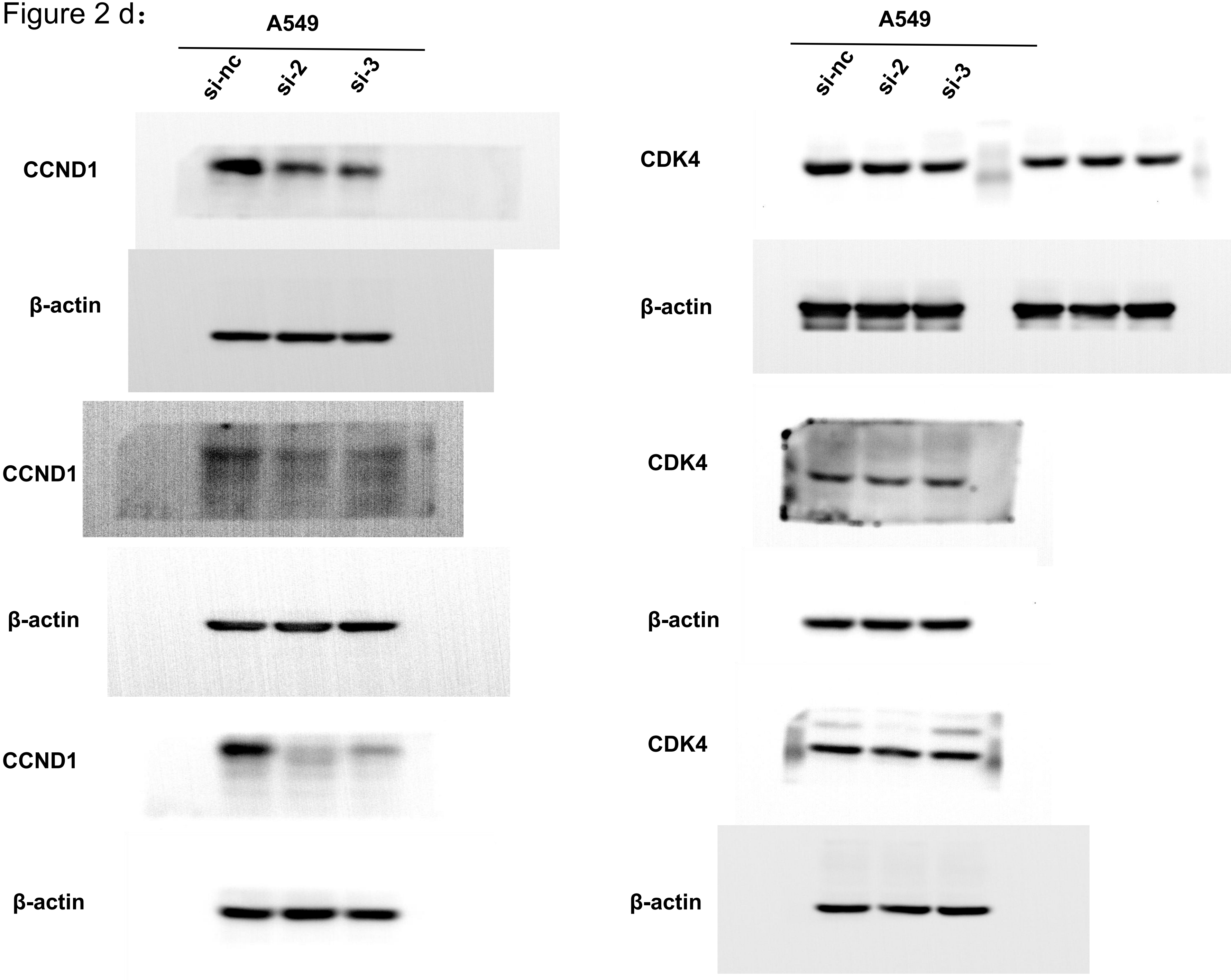
A549
si-nc
si-2
si-3
CCND1
β-actin
CCND1
β-actin
CCND1
β-actin
A549
si-nc
si-2
si-3
CDK4
β-actin
CDK4
β-actin
CDK4
β-actin

Figure 2 d:

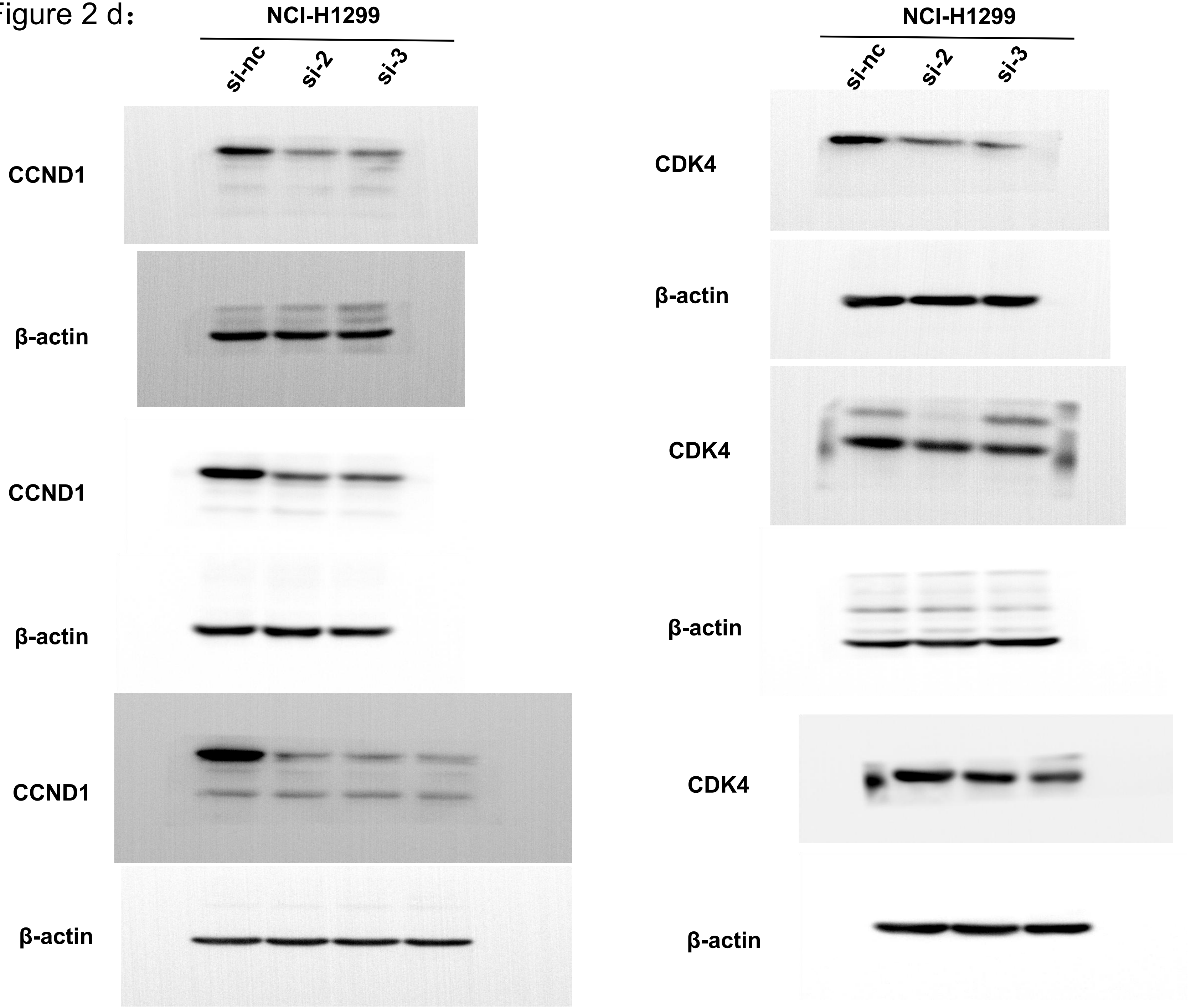

NCI-H1299
si-nc
si-2
si-3
CCND1
β-actin
CCND1
β-actin
CCND1
β-actin
NCI-H1299
si-nc
si-2
si-3
CDK4
β-actin
CDK4
β-actin
CDK4
β-actin

Figure 3 b：

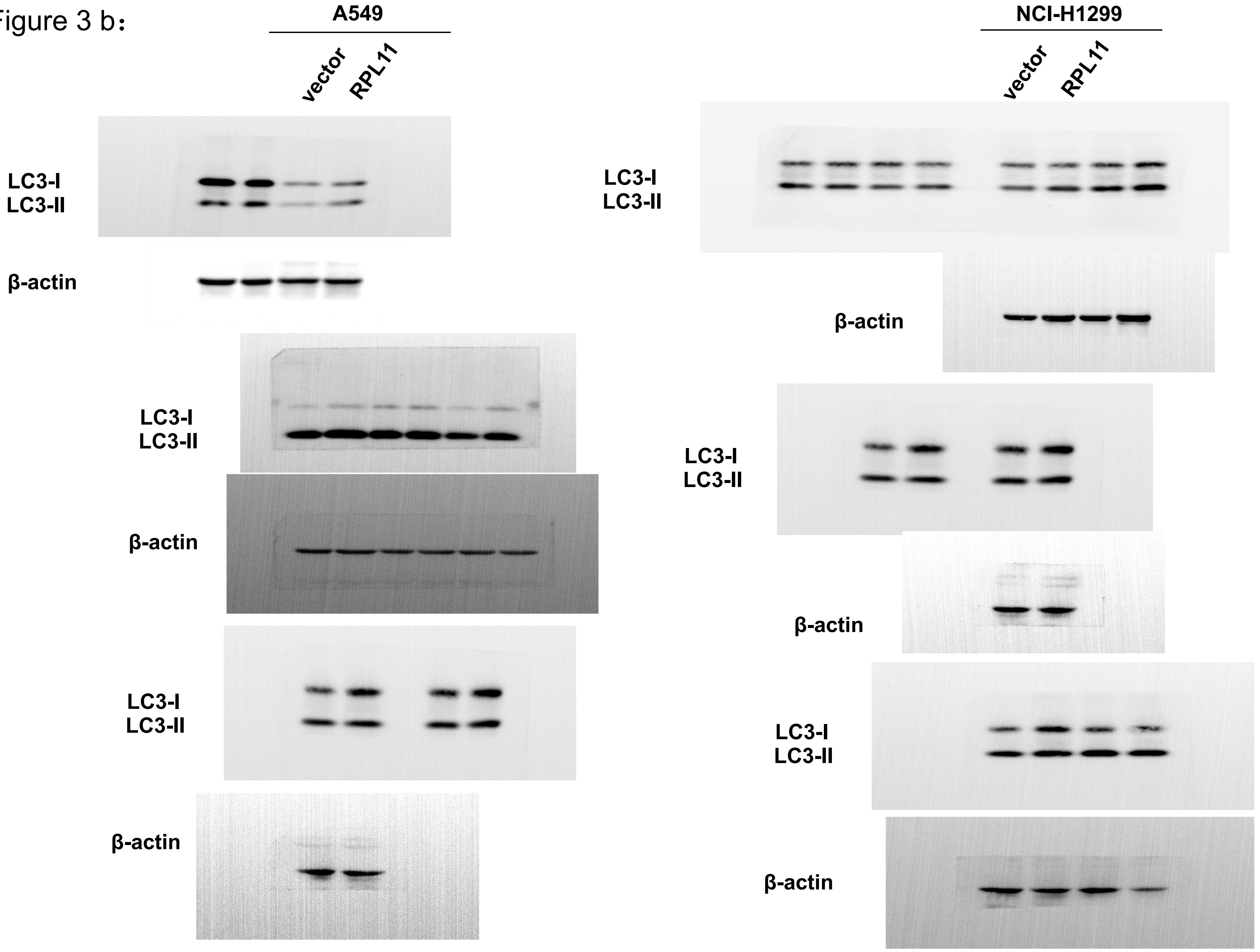
A549
vector
RPL11
LC3-I
LC3-II
β-actin
LC3-I
LC3-II
β-actin
LC3-I
LC3-II
β-actin
NCI-H1299
vector
RPL11
LC3-I
LC3-II
β-actin
LC3-I
LC3-II
β-actin
LC3-I
LC3-II
β-actin

Figure 3 b：

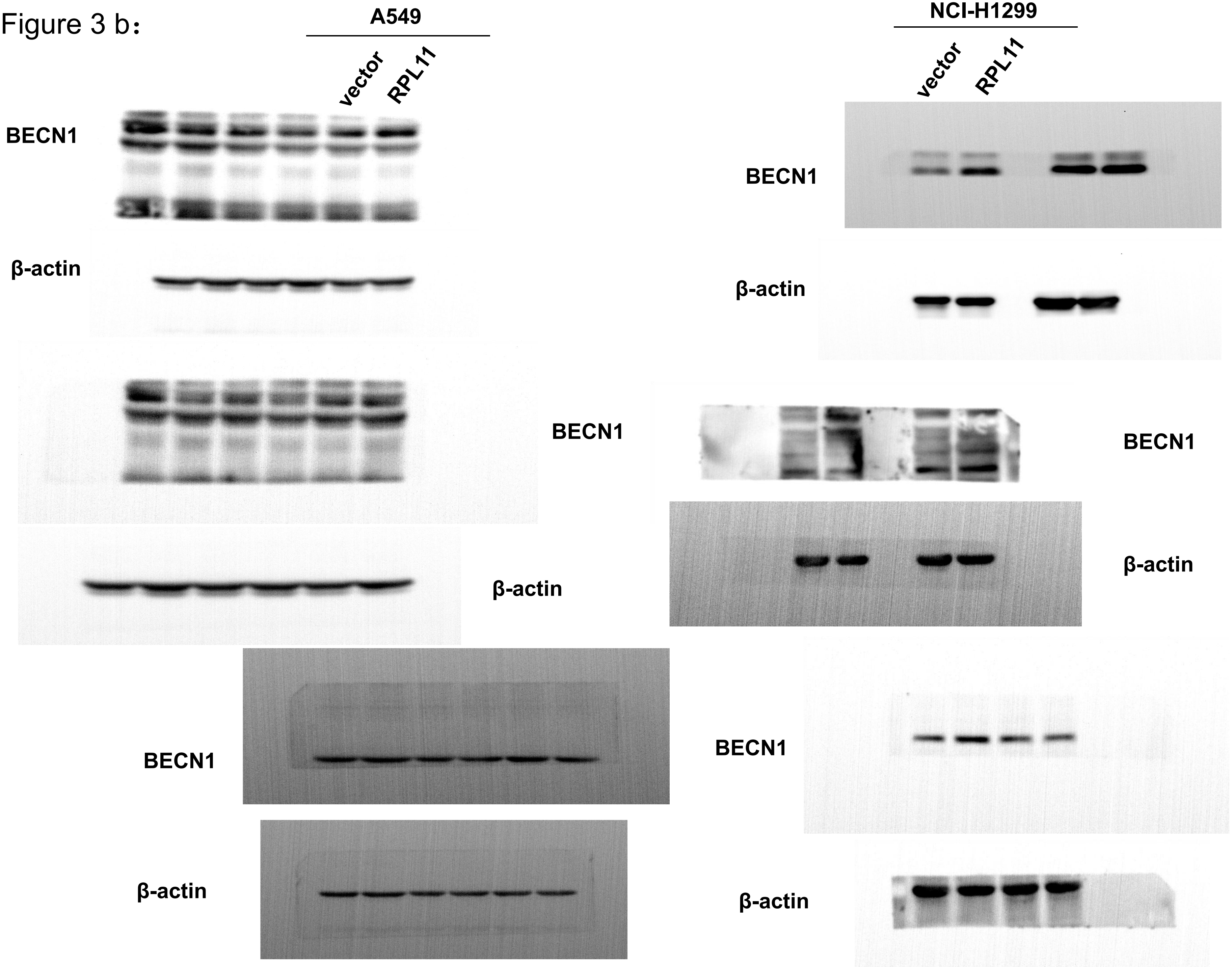
A549
vector
RPL11
BECN1
β-actin
BECN1
β-actin
BECN1
β-actin
NCI-H1299
vector
RPL11
BECN1
β-actin
BECN1
β-actin
BECN1
β-actin

Figure 3 b：

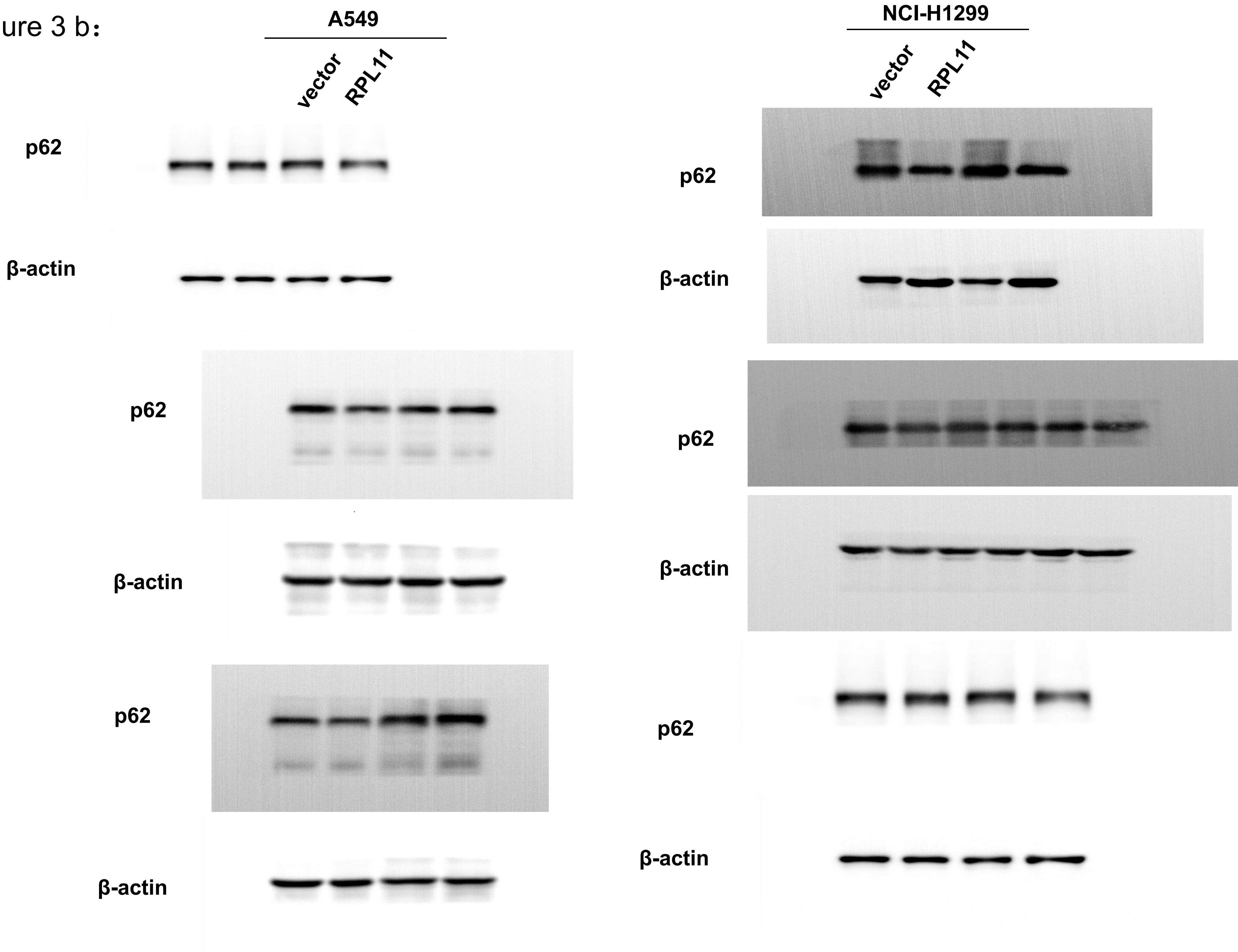
A549
vector
RPL11
p62
β-actin
p62
β-actin
p62
β-actin
NCI-H1299
vector
RPL11
p62
β-actin
p62
β-actin
p62
β-actin

Figure 3 b：

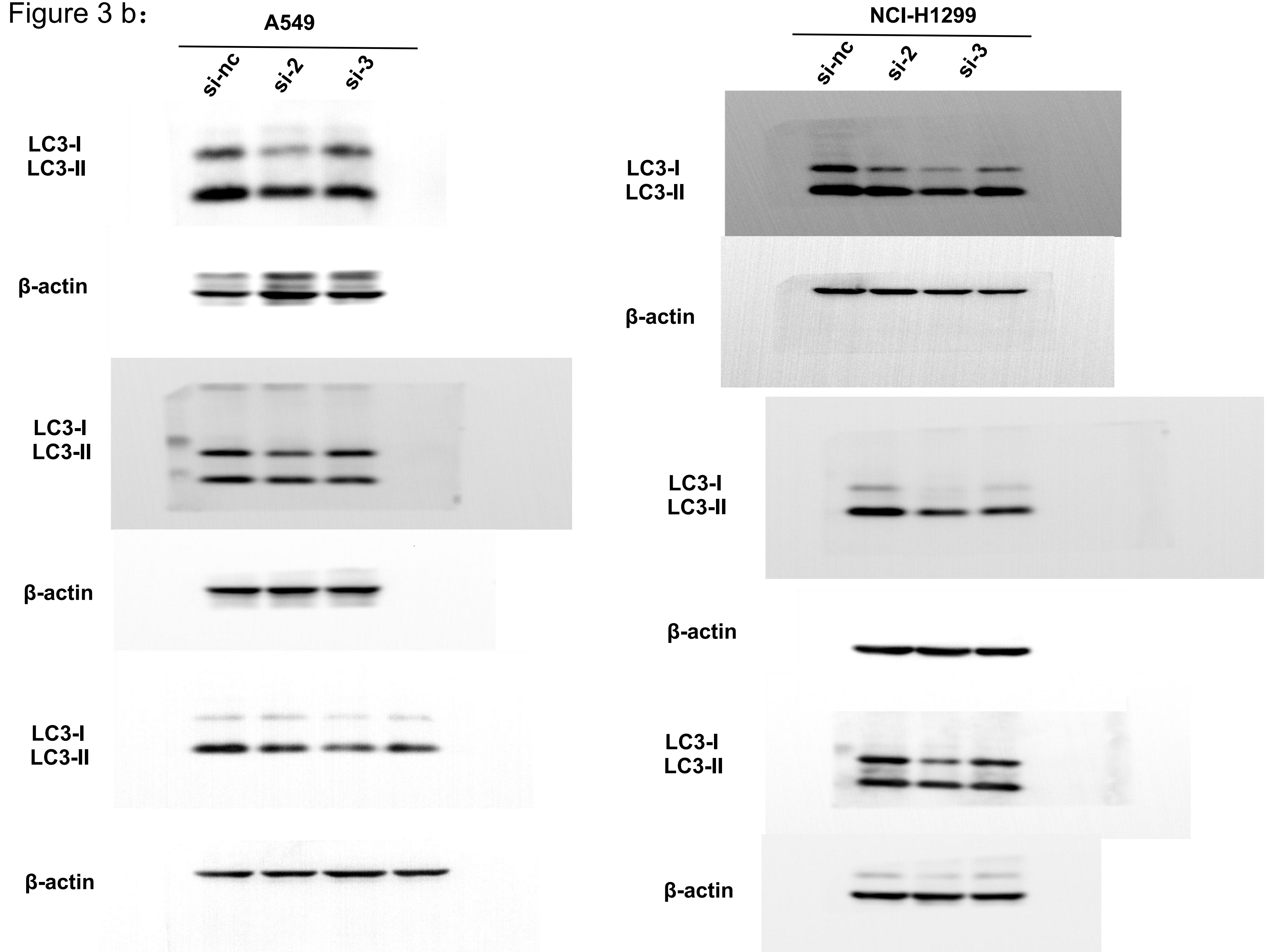
A549
si-nc
si-2
si-3
LC3-I
LC3-II
β-actin
LC3-I
LC3-II
β-actin
LC3-I
LC3-II
β-actin
NCI-H1299
si-nc
si-2
si-3
LC3-I
LC3-II
β-actin
LC3-I
LC3-II
β-actin
LC3-I
LC3-II
β-actin

Figure 3 b：

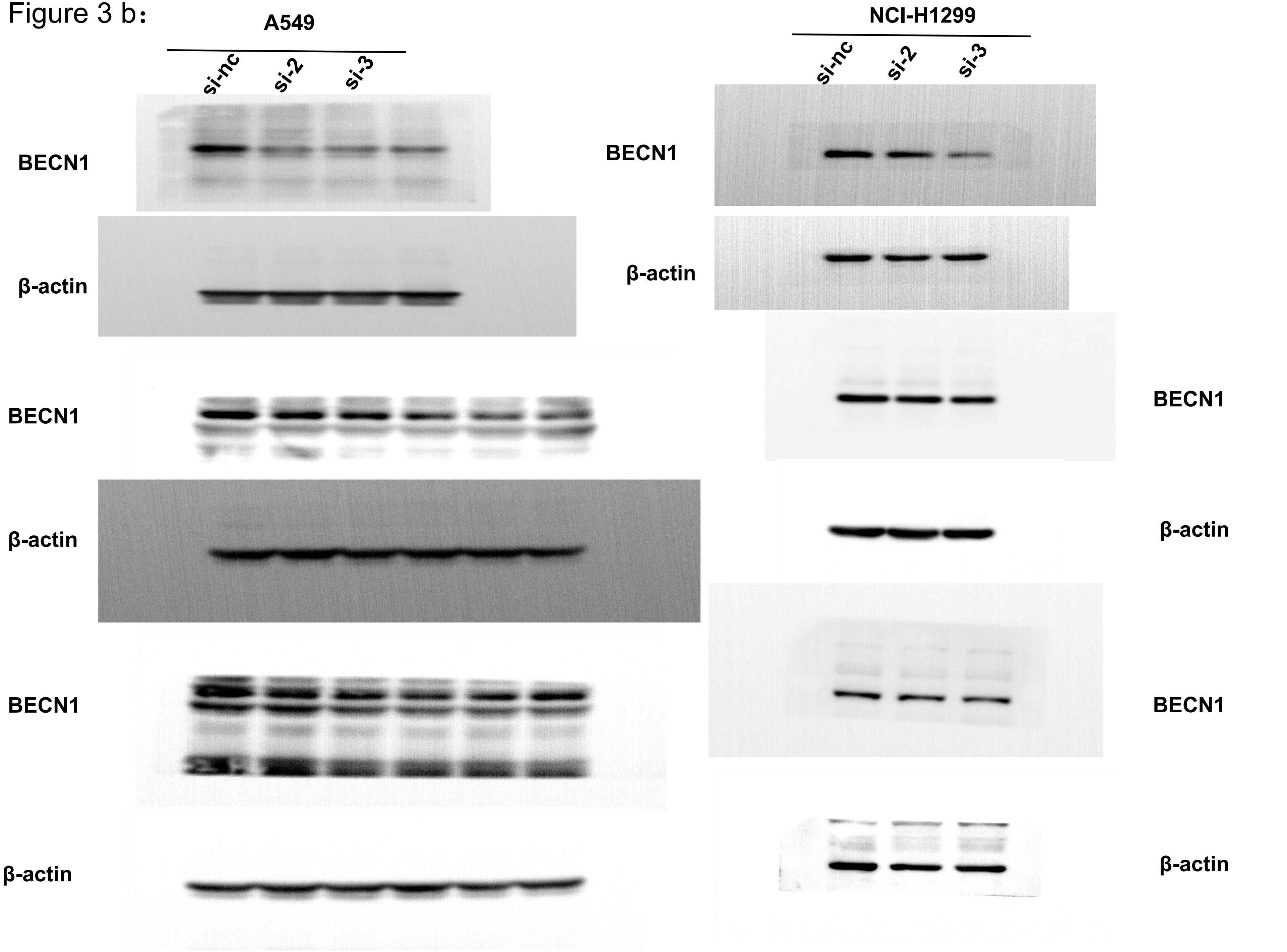
A549
si-nc
si-2
si-3
NCI-H1299
si-nc
si-2
si-3
BECN1
β-actin
BECN1
β-actin
BECN1
β-actin
BECN1
β-actin
BECN1
β-actin
BECN1
β-actin

Figure 3 b：

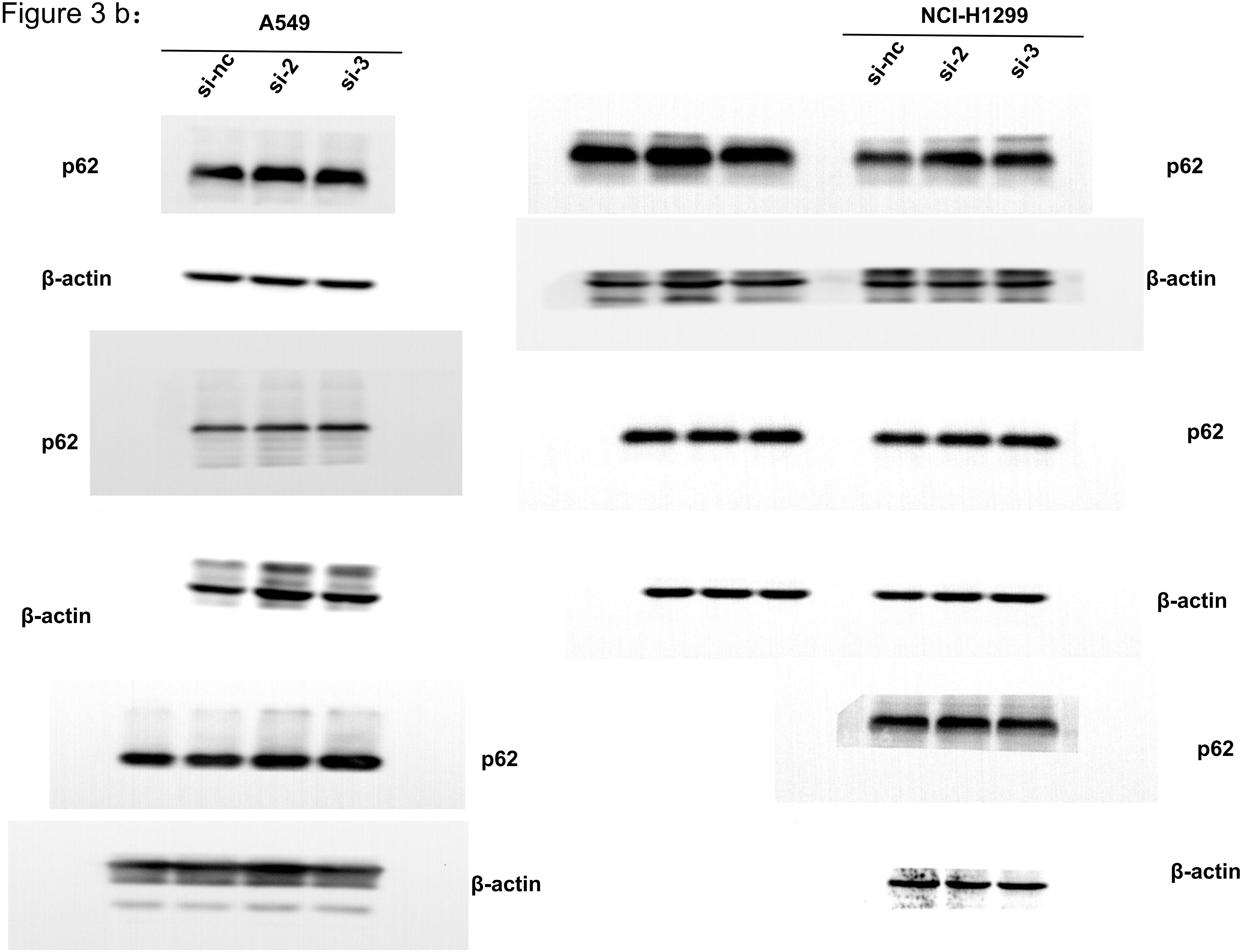
A549
si-nc
si-2
si-3
NCI-H1299
si-nc
si-2
si-3
p62
β-actin
p62
β-actin
p62
β-actin
p62
β-actin
p62
β-actin
p62
β-actin

Figure 3 f:

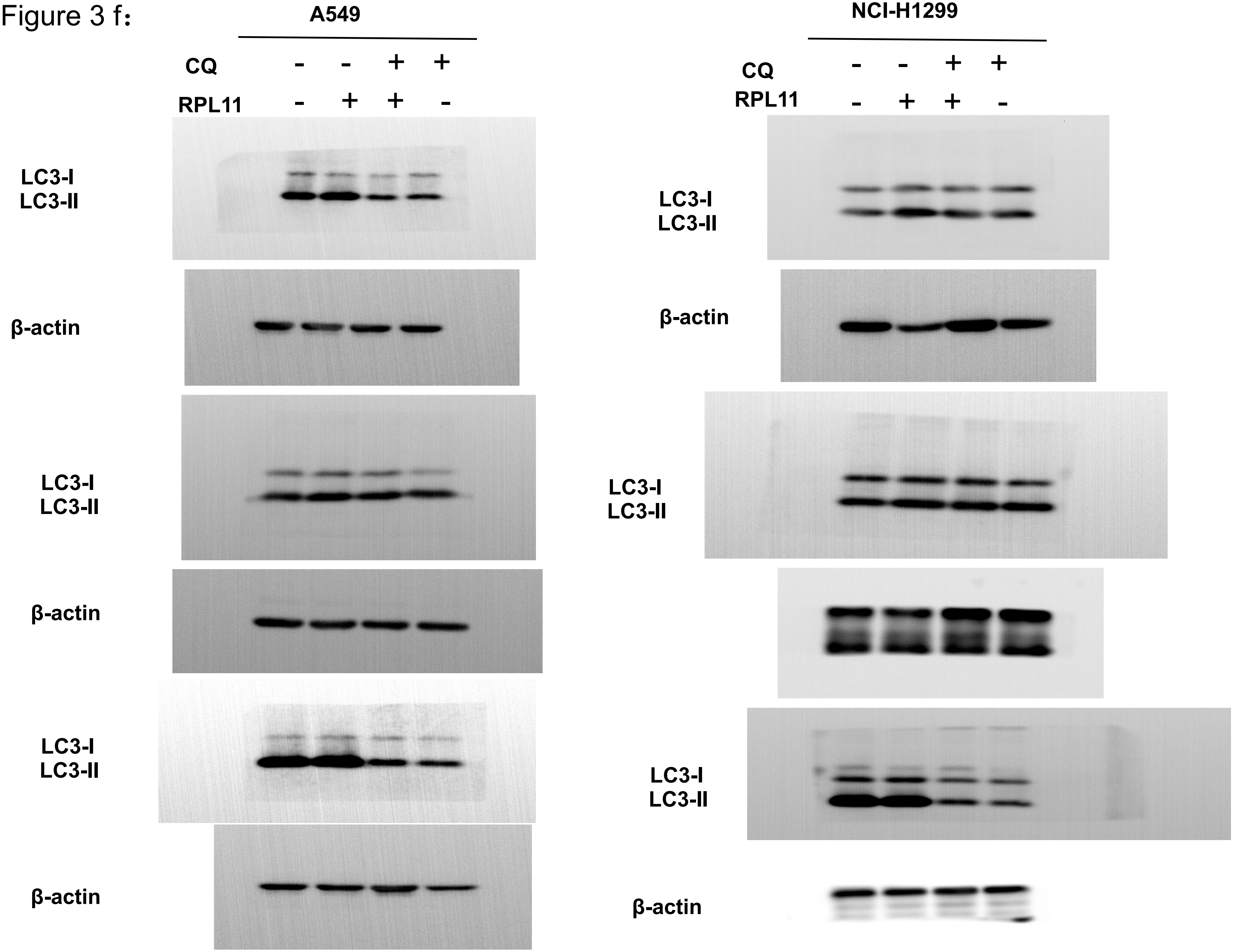
A549
CQ - - + +
RPL11 - + + -
LC3-I
LC3-II
β-actin
LC3-I
LC3-II
β-actin
LC3-I
LC3-II
β-actin
NCI-H1299
CQ - - + +
RPL11 - + + -
LC3-I
LC3-II
β-actin
LC3-I
LC3-II
LC3-I
LC3-II
β-actin

Figure 3 f:

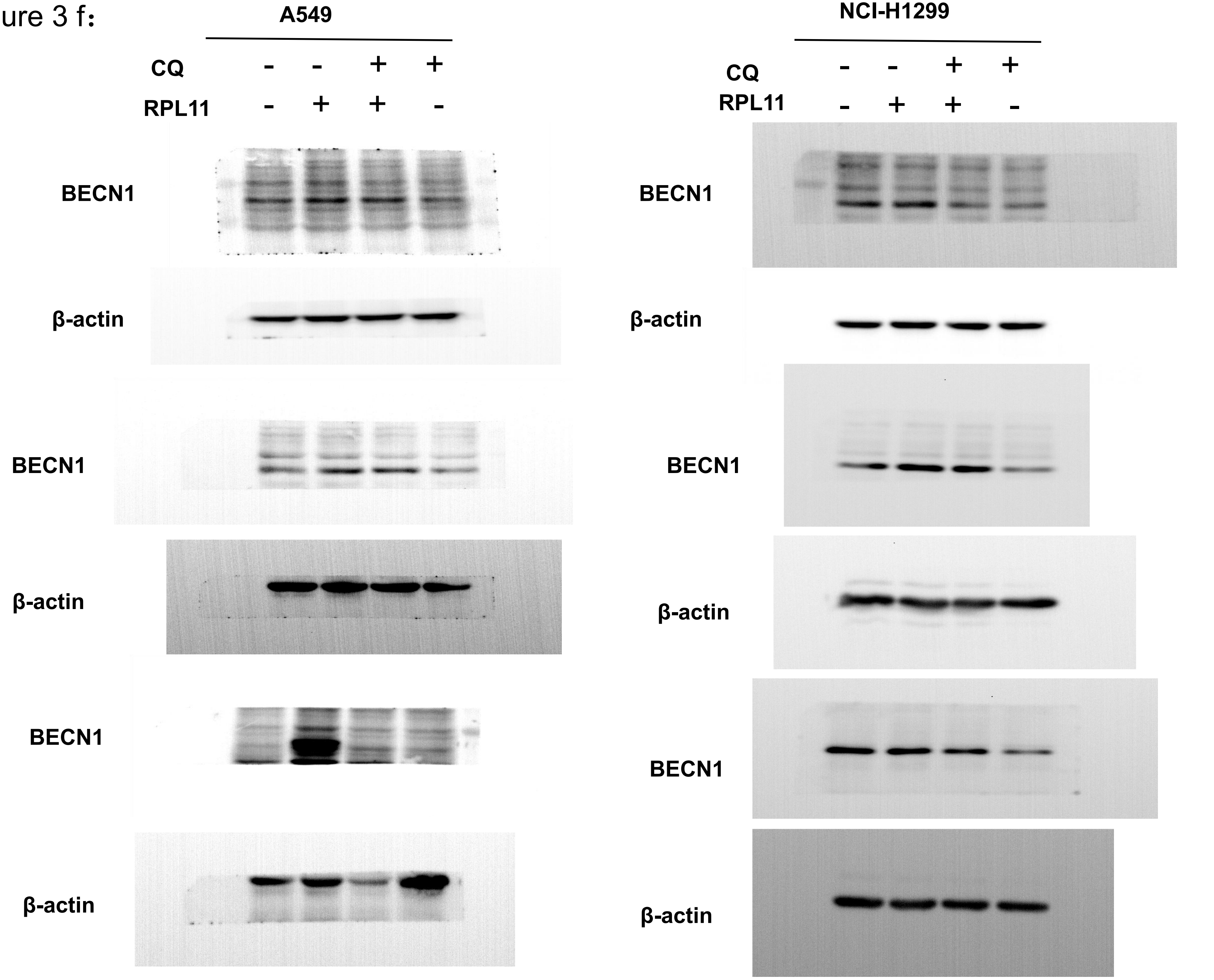
A549
NCI-H1299
CQ - - + +
RPL11 - + + -
BECN1
β-actin
BECN1
β-actin
BECN1
β-actin
CQ - - + +
RPL11 - + + -
BECN1
β-actin
BECN1
β-actin
BECN1
β-actin

Figure 3 f：

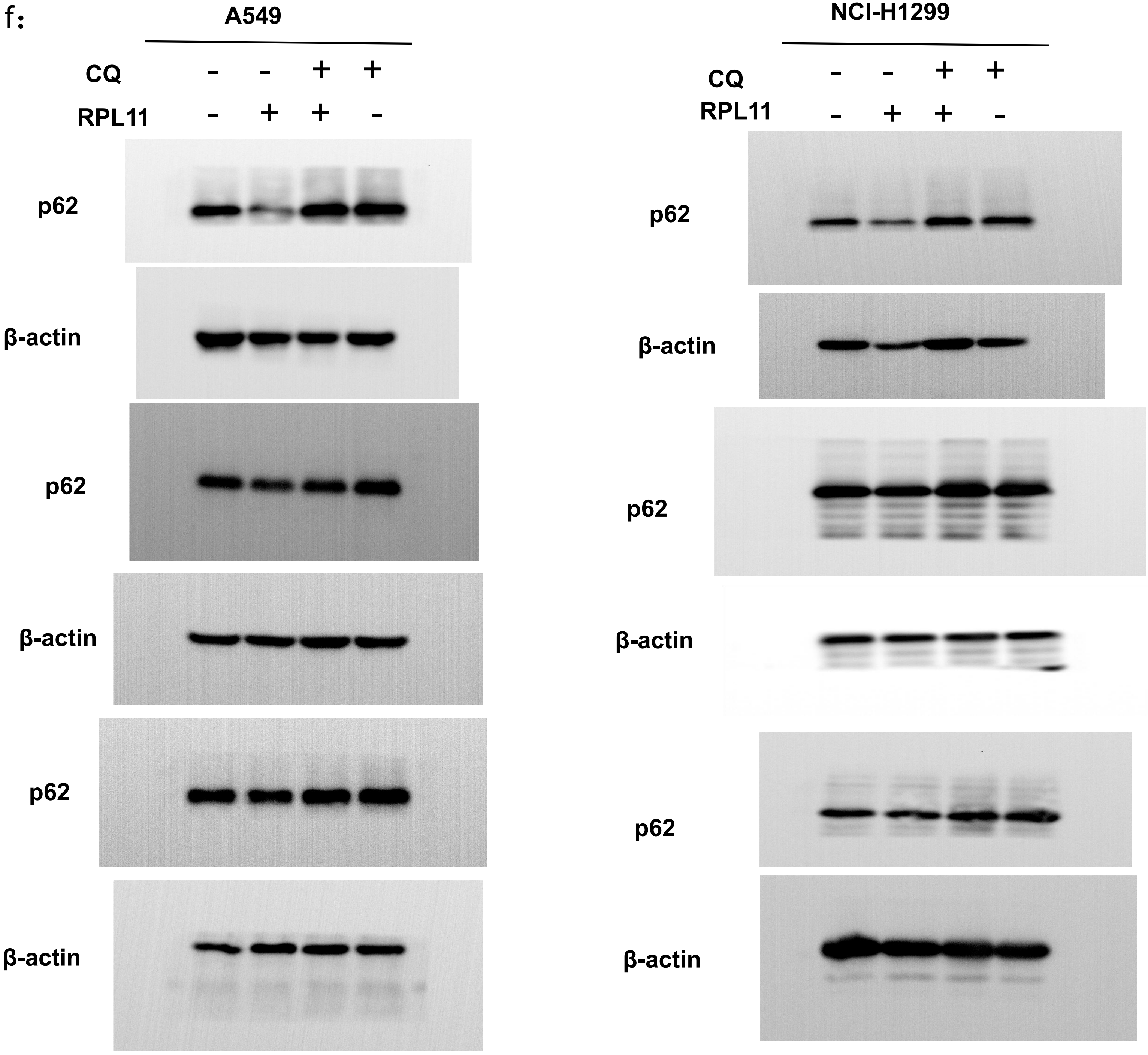
A549
CQ - - + +
RPL11 - + + -
p62
β-actin
p62
β-actin
p62
β-actin
NCI-H1299
CQ - - + +
RPL11 - + + -
p62
β-actin
p62
β-actin
p62
β-actin

Figure 3 f:

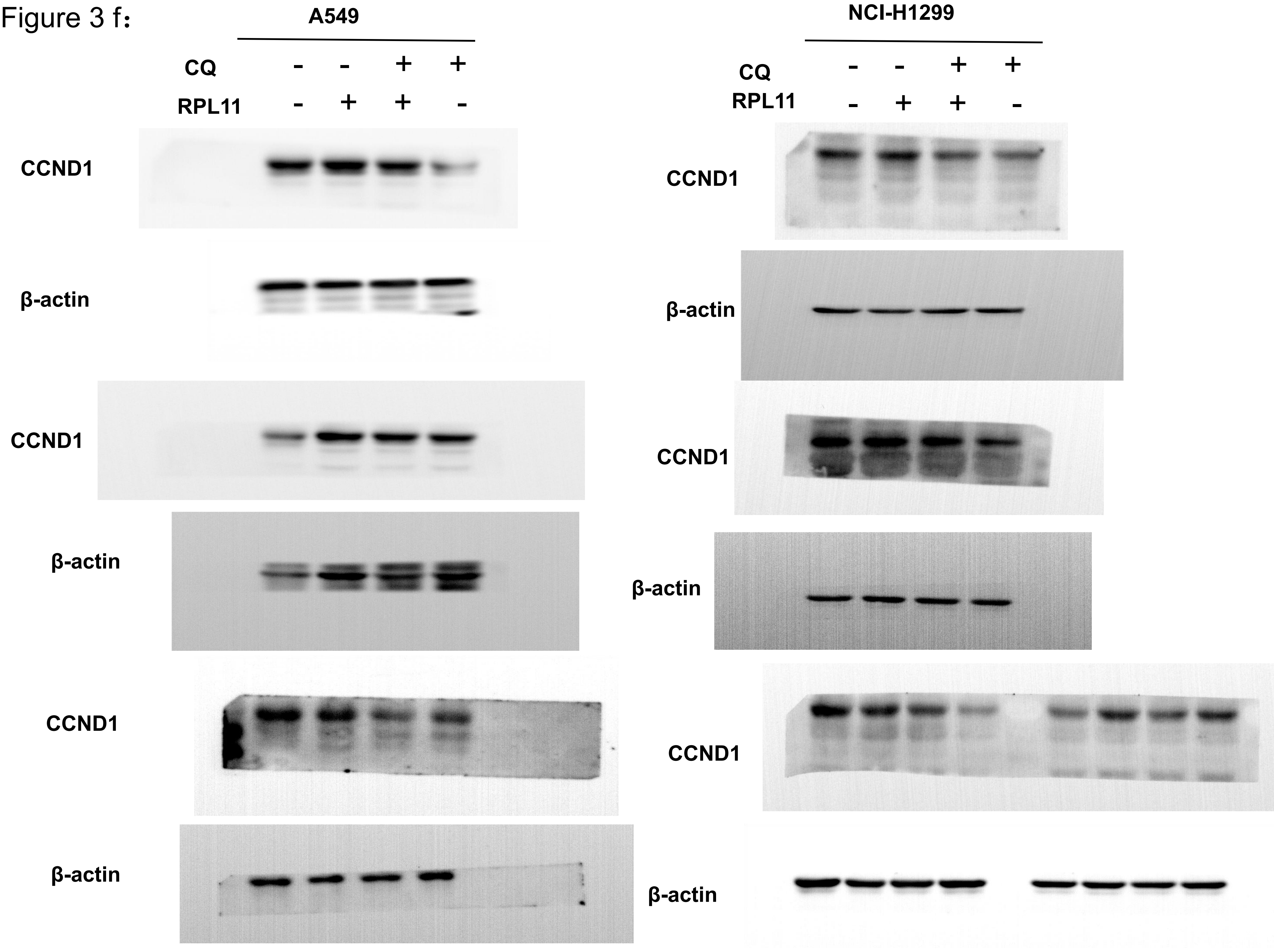
A549
CQ - - + +
RPL11 - + + -
CCND1
β-actin
CCND1
β-actin
CCND1
β-actin
NCI-H1299
CQ - - + +
RPL11 - + + -
CCND1
β-actin
CCND1
β-actin
CCND1
β-actin

Figure 3 f:

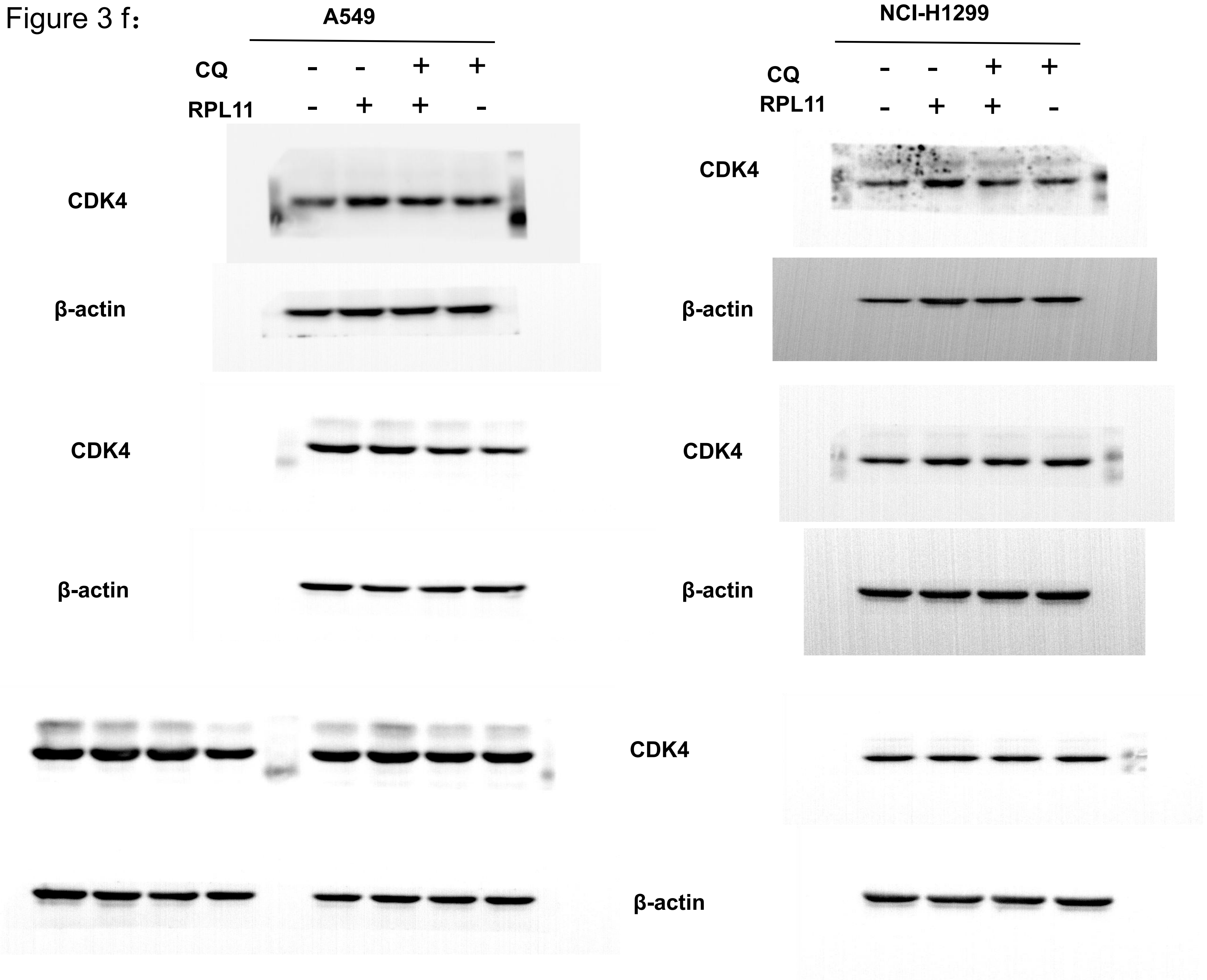
A549
NCI-H1299
CQ - - + +
RPL11 - + + -
CQ - - + +
RPL11 - + + -
CDK4
β-actin
CDK4
β-actin
CDK4
β-actin
CDK4
β-actin
CDK4
β-actin

Figure 4 a:

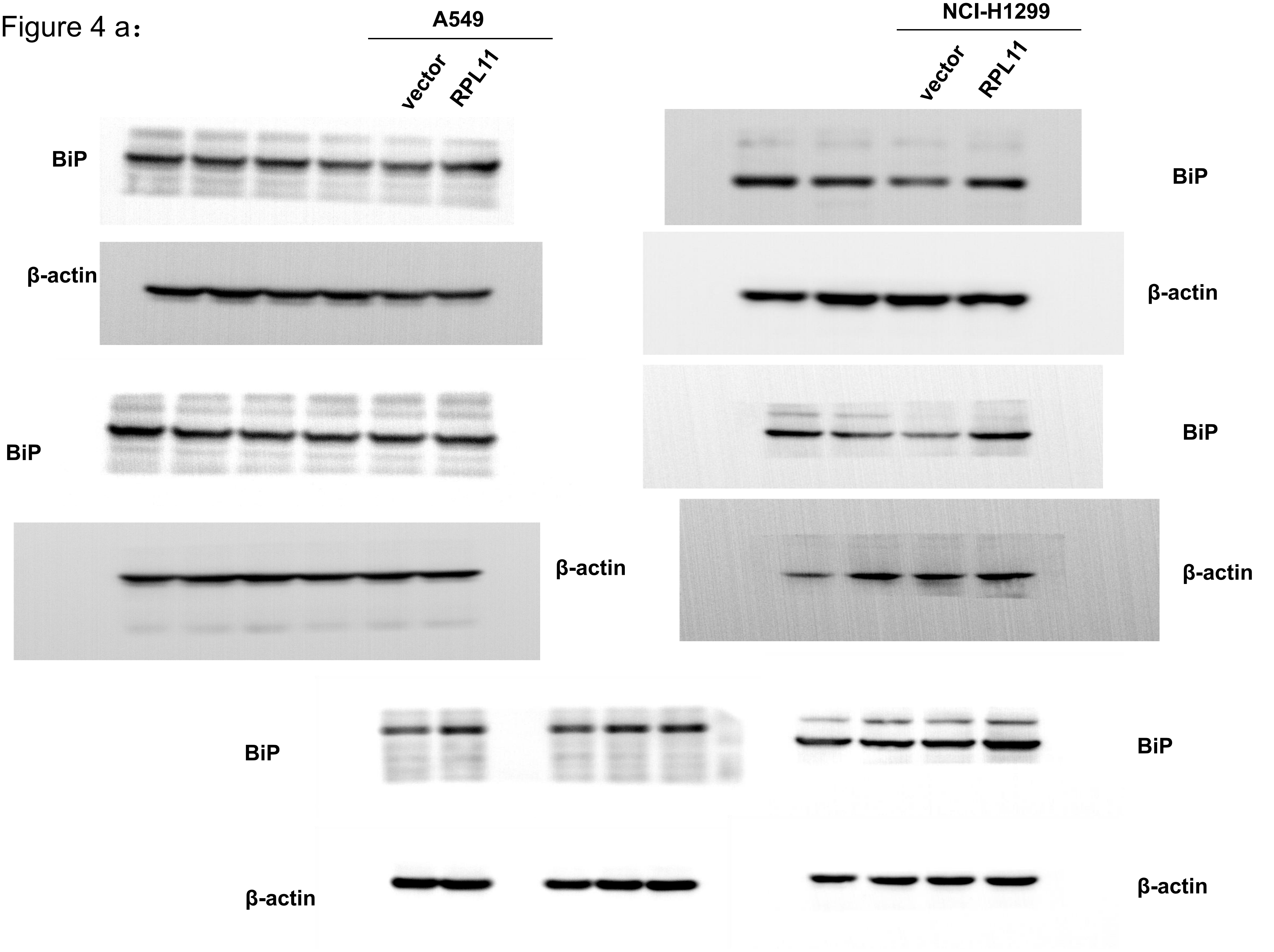
A549
vector
RPL11
NCI-H1299
vector
RPL11
BiP
β-actin
BiP
β-actin
BiP
β-actin
BiP
β-actin
BiP
β-actin
BiP
β-actin

Figure 4 a：

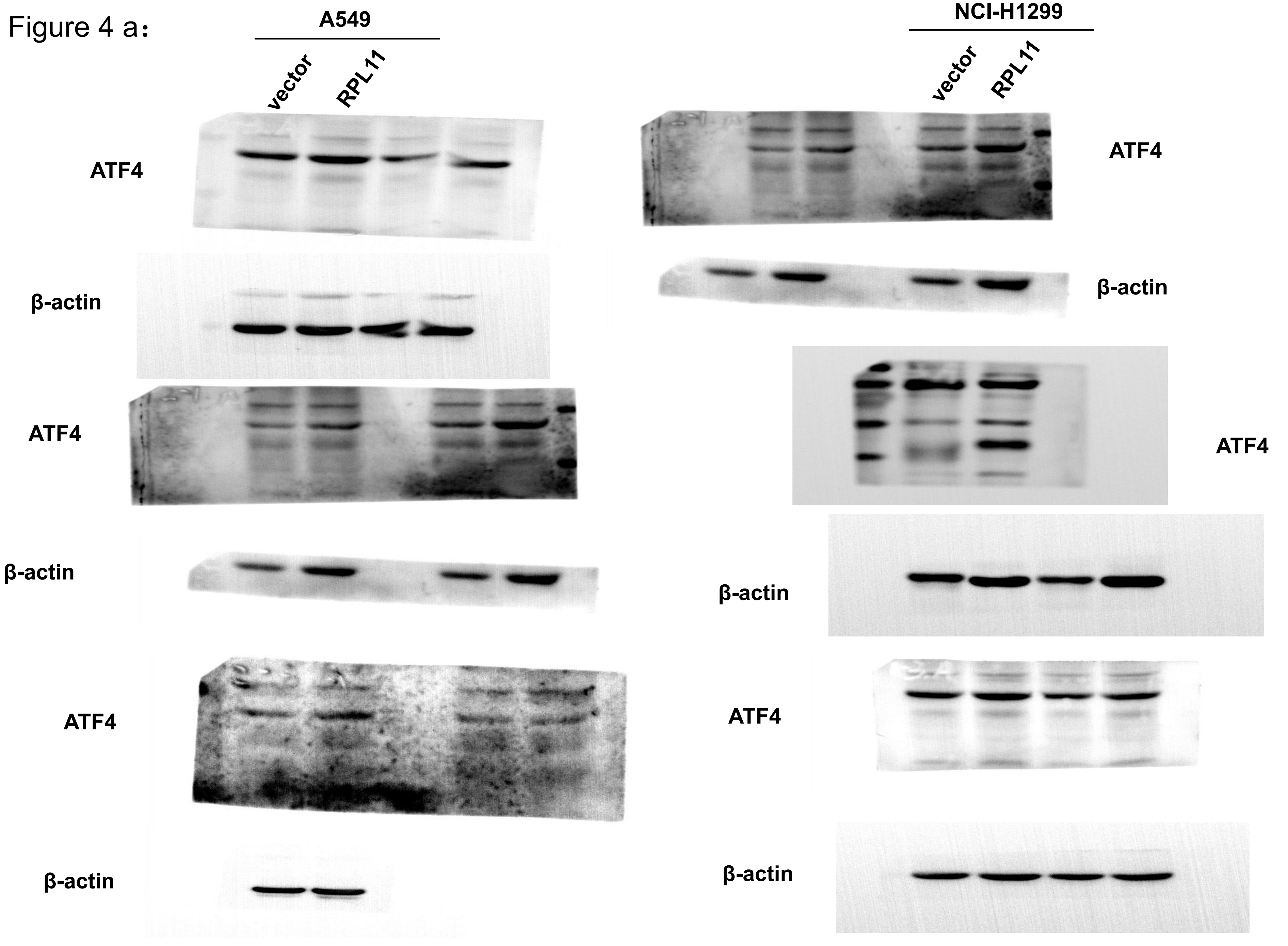
A549
vector
RPL11
NCI-H1299
vector
RPL11
ATF4
β-actin
ATF4
β-actin
ATF4
β-actin
ATF4
β-actin
ATF4
β-actin
ATF4
β-actin

Figure 4 a:

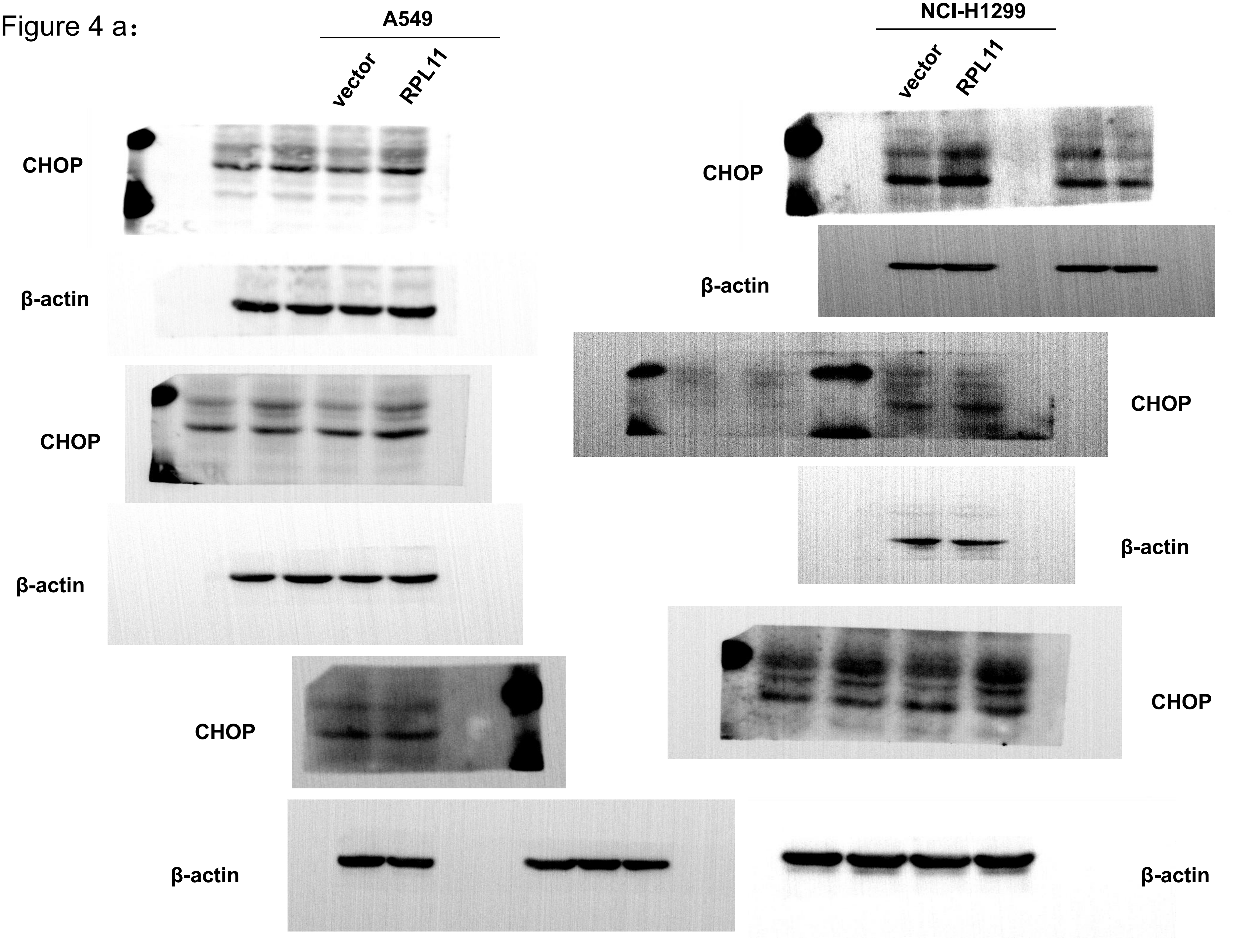
A549
vector
RPL11
NCI-H1299
vector
RPL11
CHOP
β-actin
CHOP
β-actin
CHOP
β-actin
CHOP
β-actin
CHOP
β-actin
CHOP
β-actin

Figure 4 a:

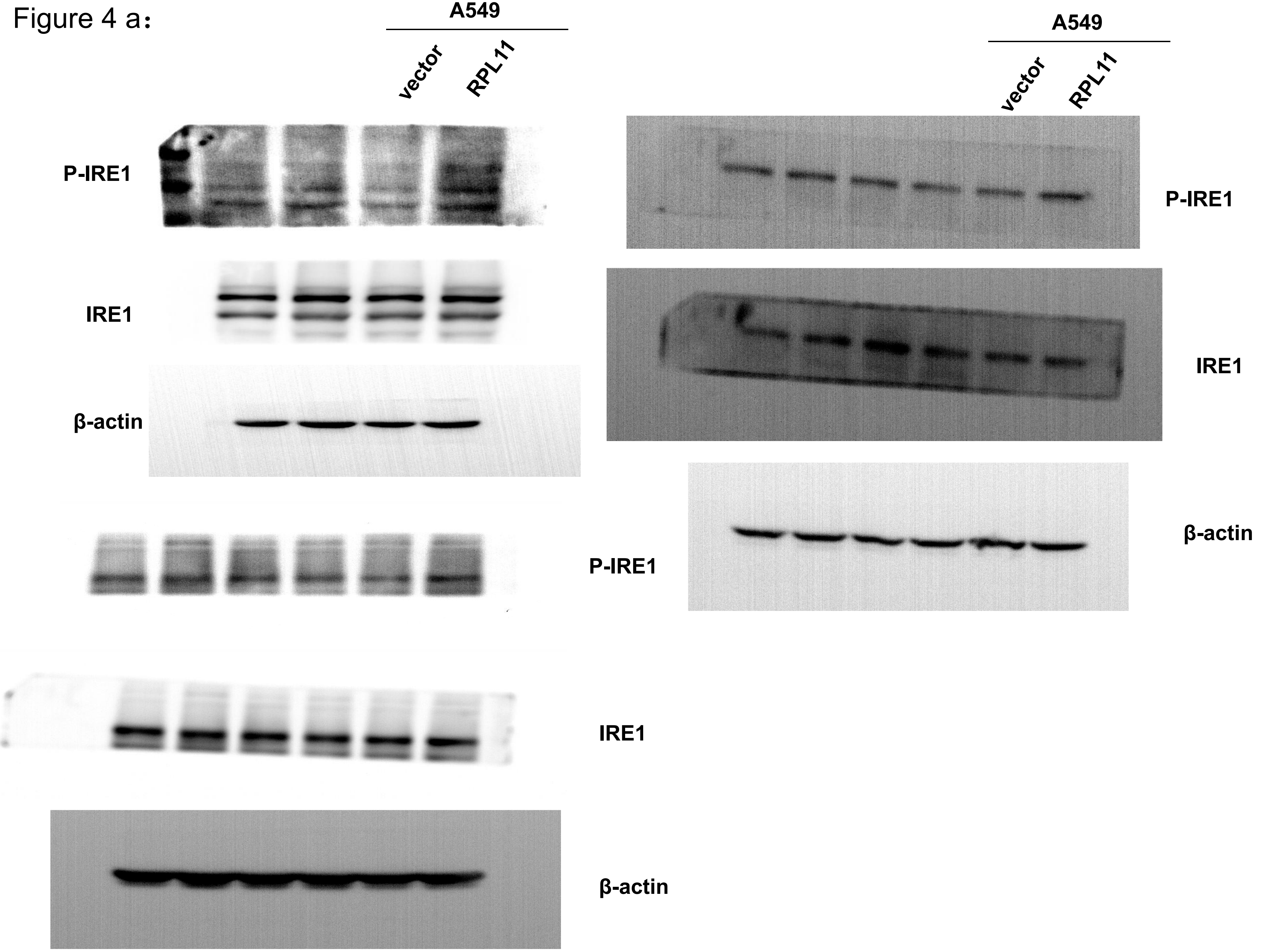
A549
vector
RPL11
P-IRE1
IRE1
β-actin
P-IRE1
IRE1
β-actin
A549
vector
RPL11
P-IRE1
IRE1
β-actin

Figure 4 a：

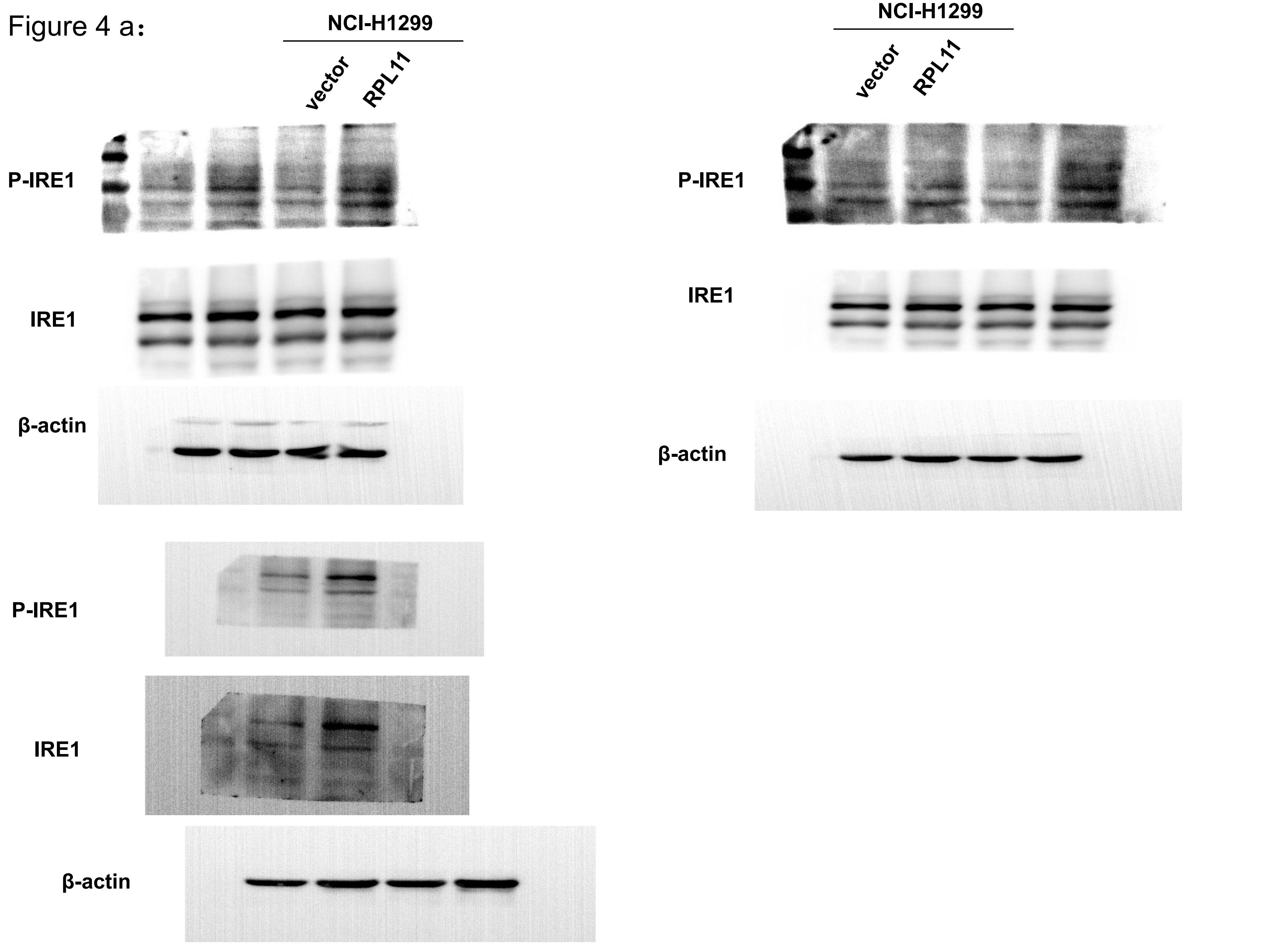
NCI-H1299
vector
RPL11
P-IRE1
IRE1
β-actin
NCI-H1299
vector
RPL11
P-IRE1
IRE1
β-actin
P-IRE1
IRE1
β-actin

Figure 4 b：

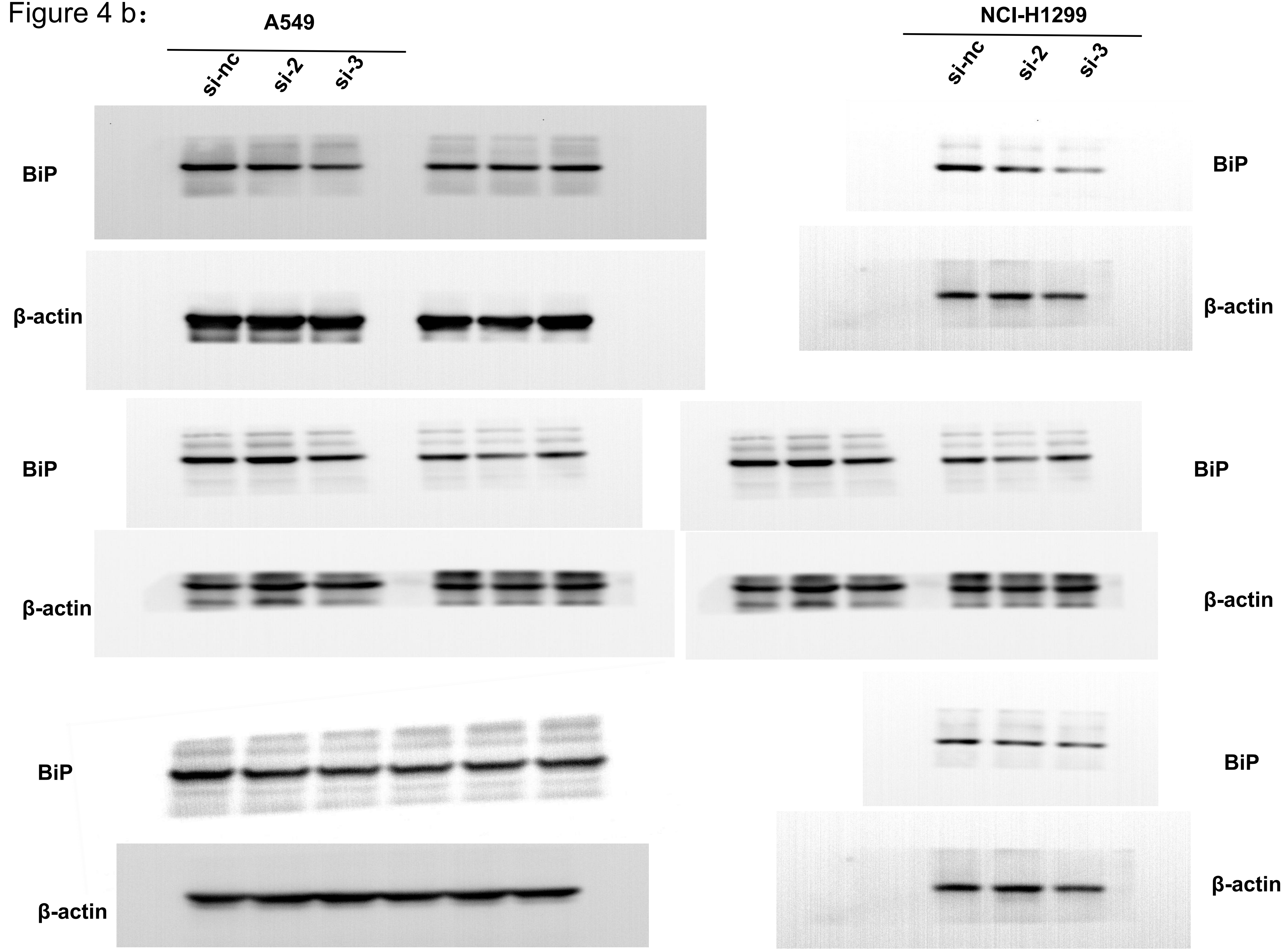
A549
si-nc
si-2
si-3
NCI-H1299
si-nc
si-2
si-3
BiP
β-actin
BiP
β-actin
BiP
β-actin
BiP
β-actin
BiP
β-actin
BiP
β-actin

Figure 4 b：

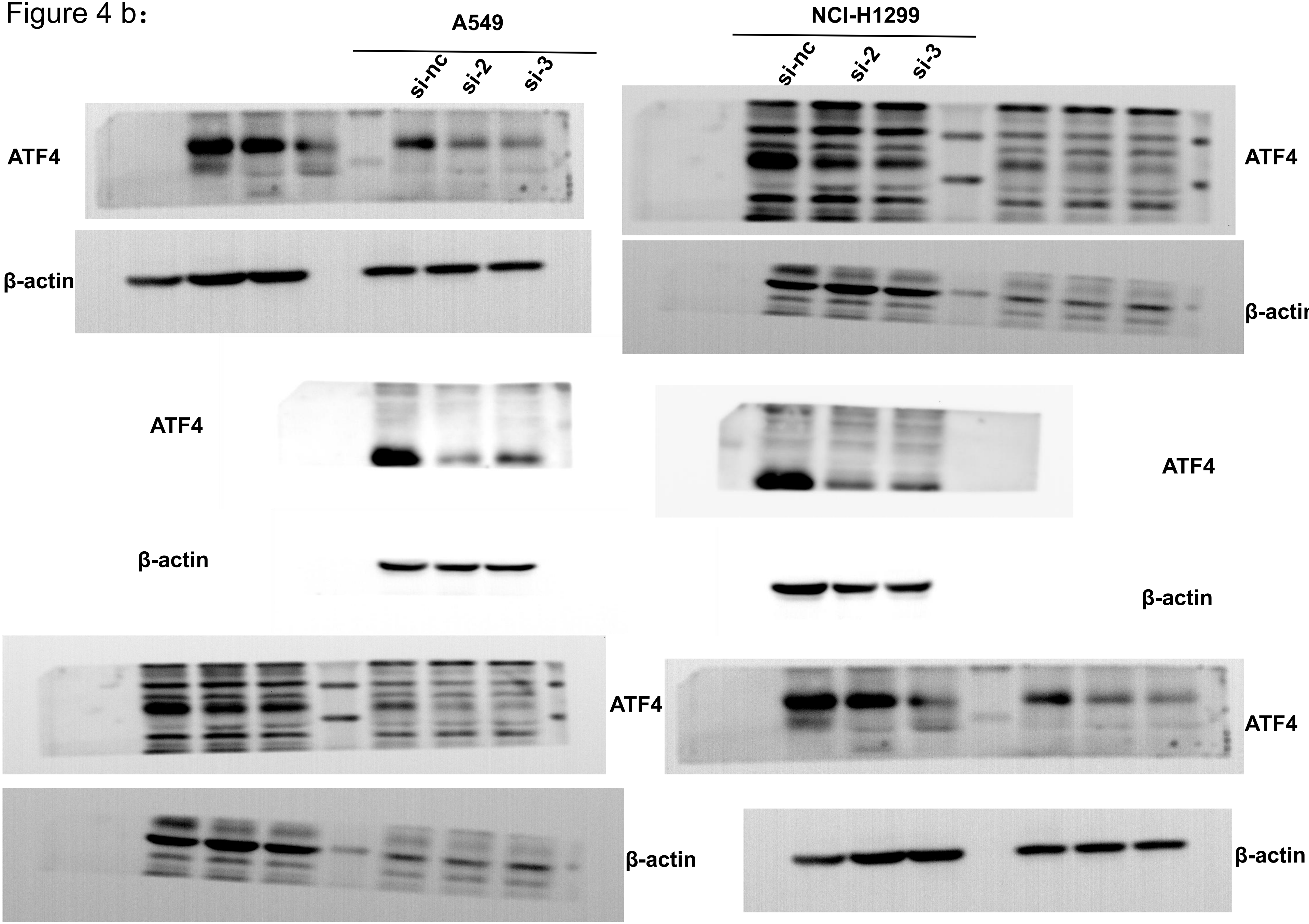
A549
si-nc
si-2
si-3
NCI-H1299
si-nc
si-2
si-3
ATF4
β-actin
ATF4
β-actin
ATF4
β-actin
ATF4
β-actin
ATF4
β-actin
ATF4
β-actin

Figure 4 b:

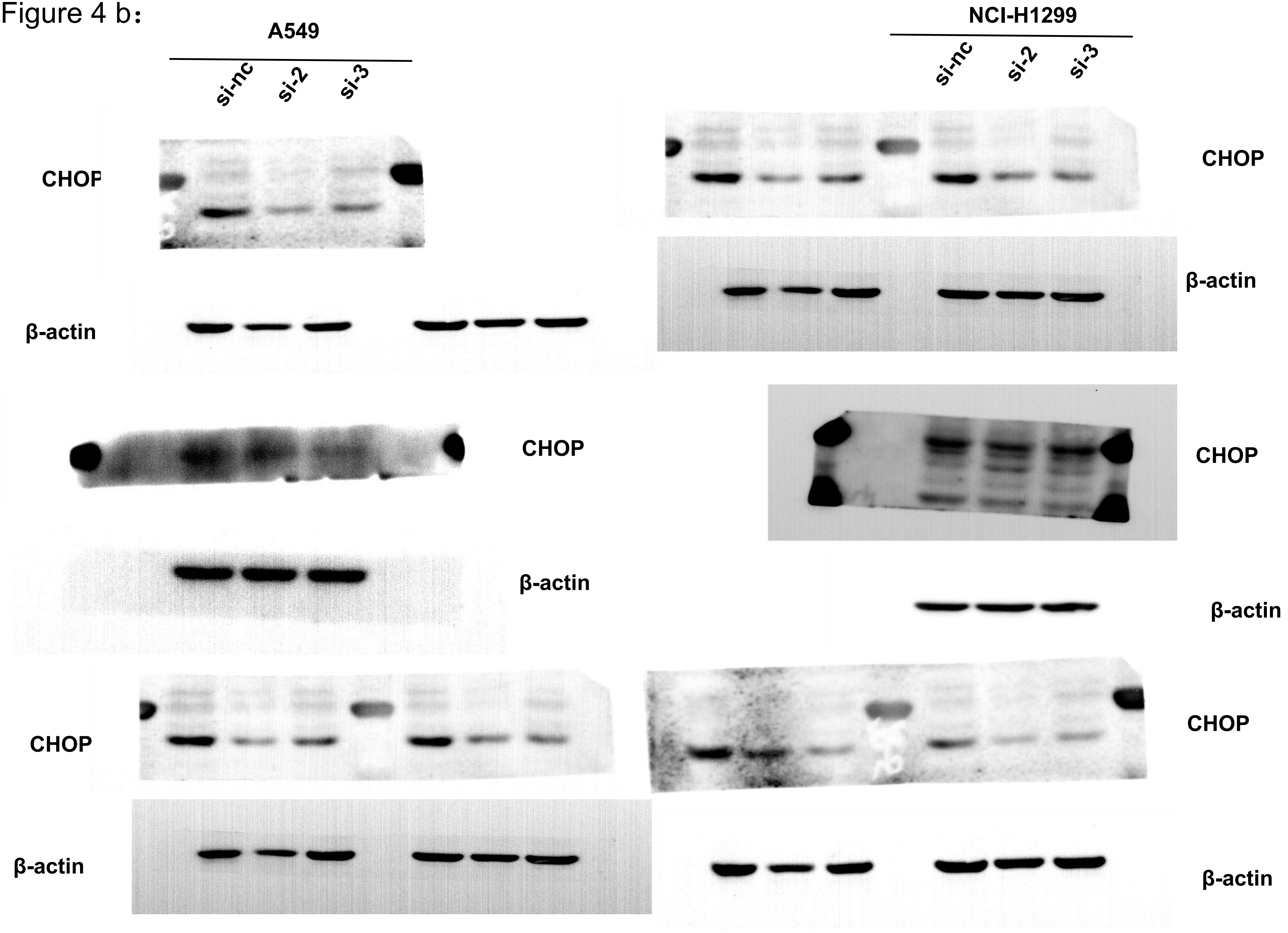
A549
si-nc
si-2
si-3
NCI-H1299
si-nc
si-2
si-3
CHOP
β-actin
CHOP
β-actin
CHOP
β-actin
CHOP
β-actin
CHOP
β-actin
CHOP
β-actin

Figure 4 b：

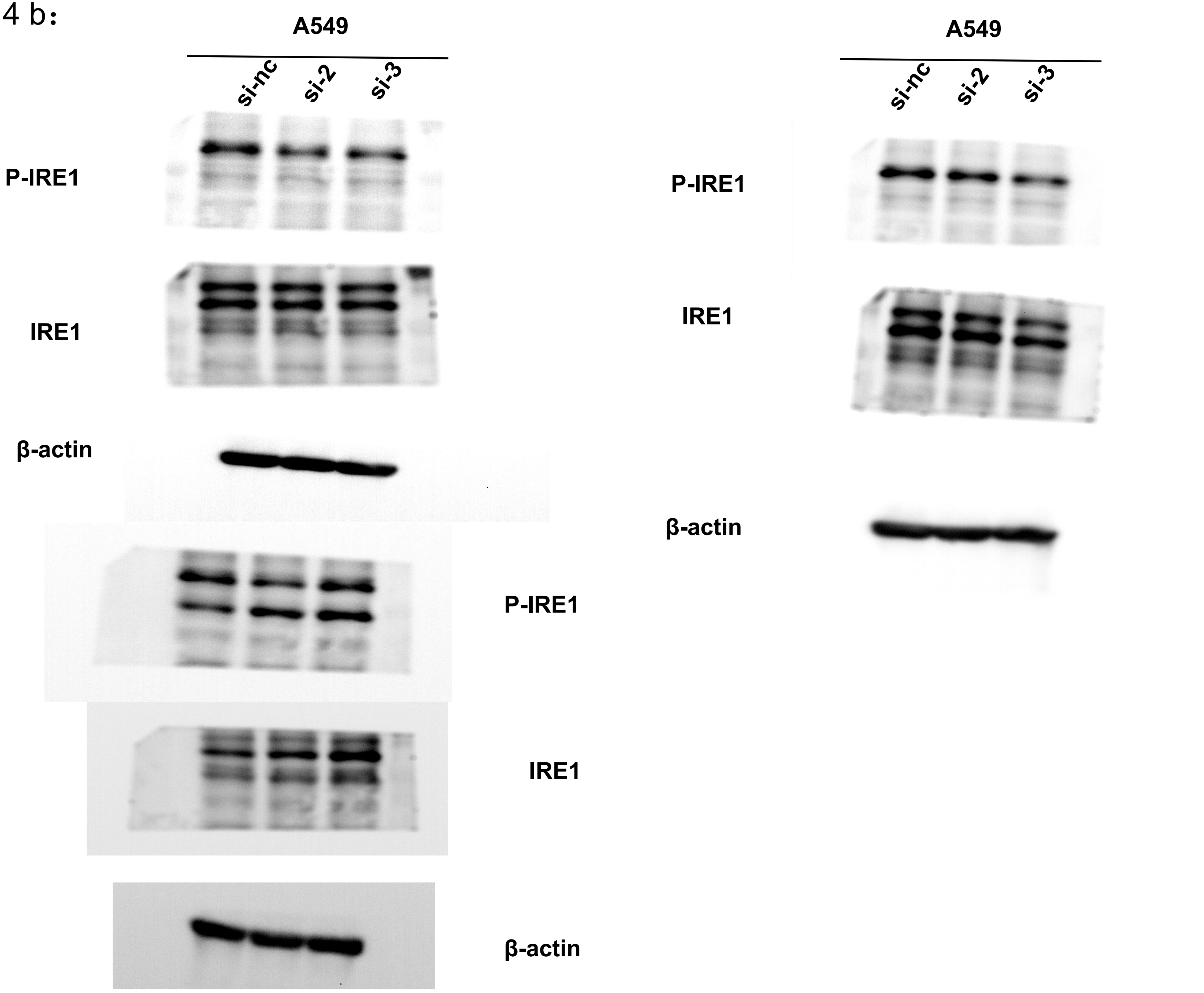
A549
si-nc
si-2
si-3
P-IRE1
IRE1
β-actin
P-IRE1
IRE1
β-actin
A549
si-nc
si-2
si-3
P-IRE1
IRE1
β-actin

Figure 4 b：

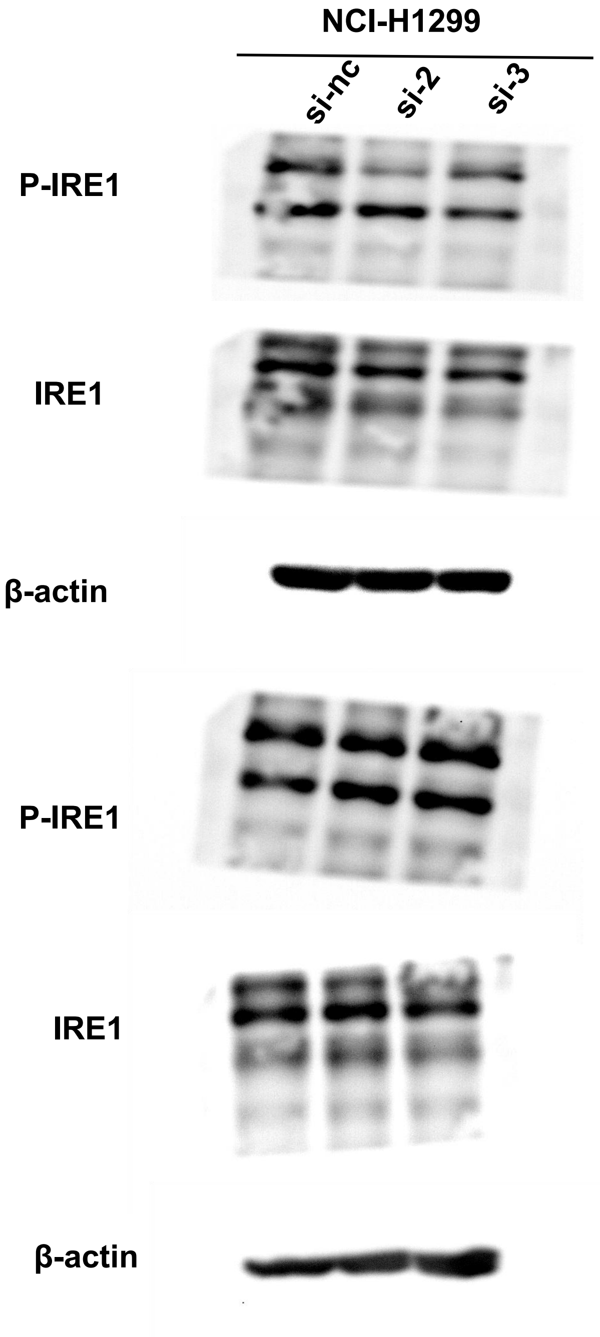
NCI-H1299
si-nc
si-2
si-3
P-IRE1
IRE1
β-actin
P-IRE1
IRE1
β-actin

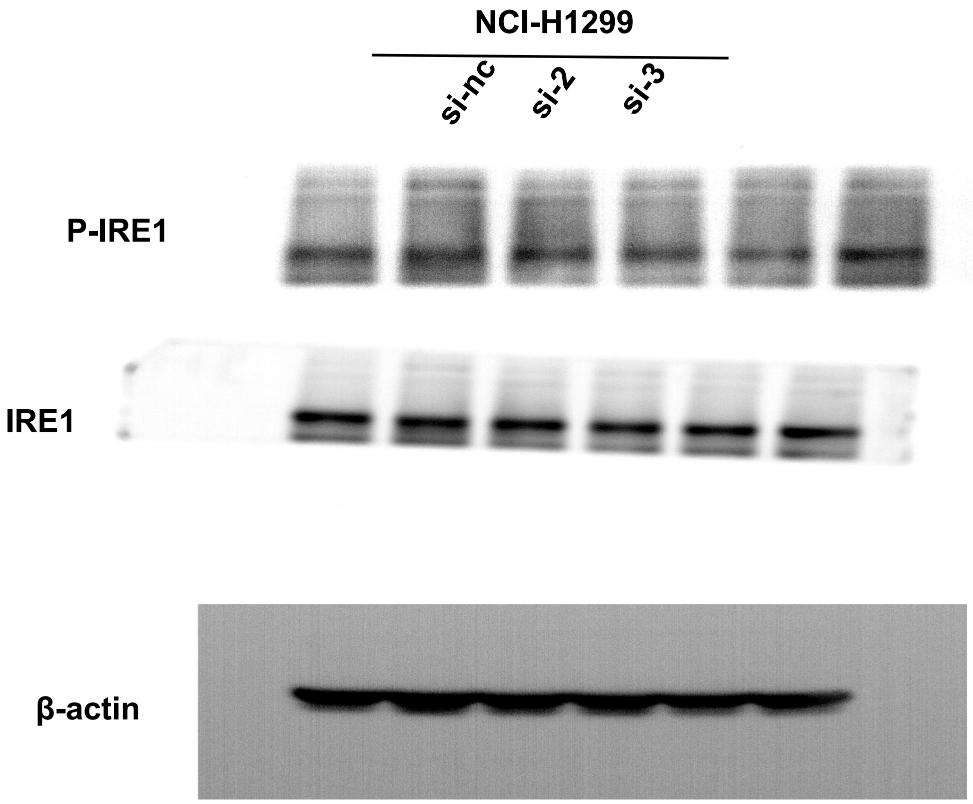
NCI-H1299
si-nc
si-2
si-3
P-IRE1
IRE1
β-actin

Figure 4 c:

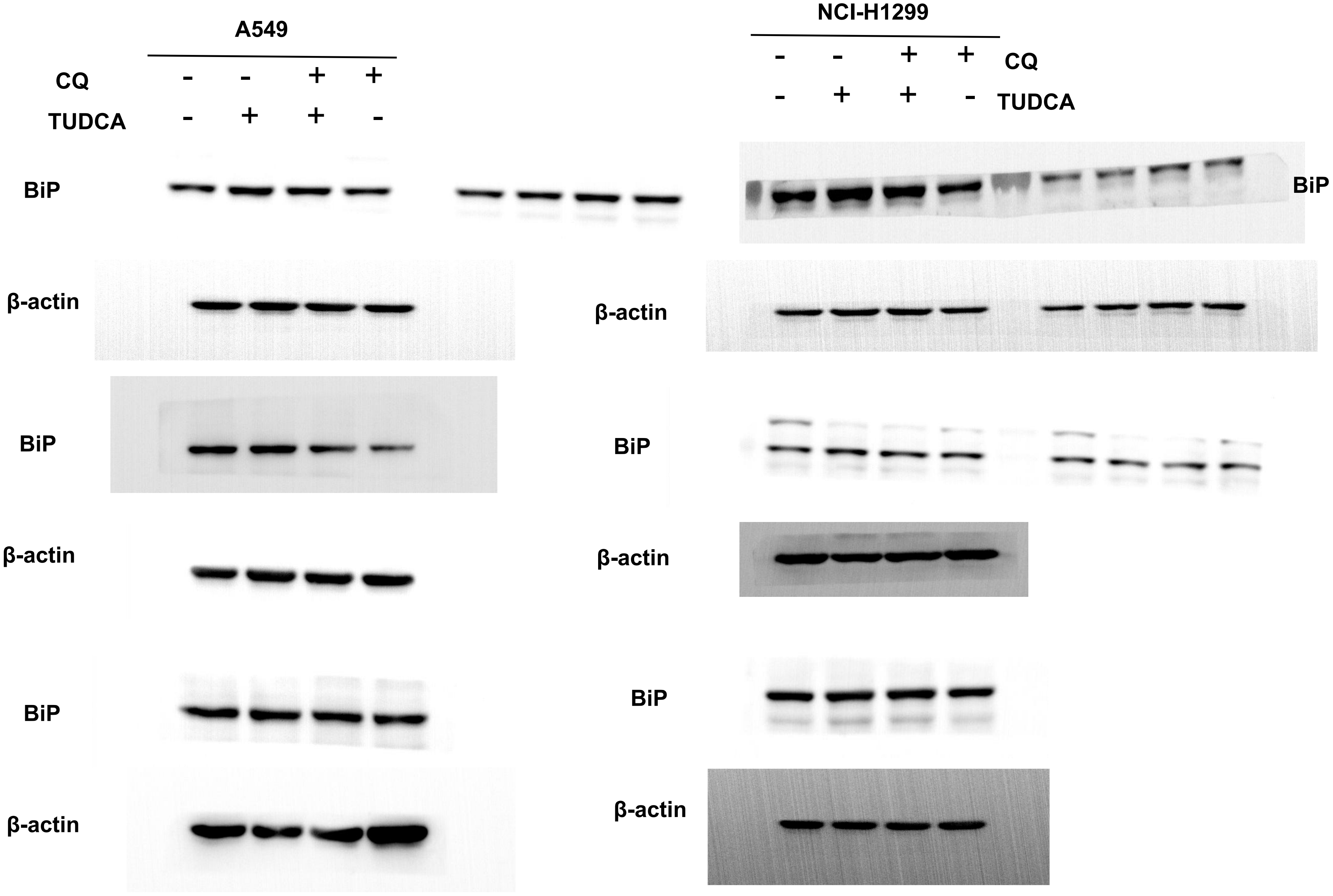
A549
CQ - - + +
TUDCA - + + -
BiP
β-actin
BiP
β-actin
BiP
β-actin
NCI-H1299
- - + + CQ
- + + - TUDCA
BiP
β-actin
BiP
β-actin
BiP
β-actin

Figure 4 c:

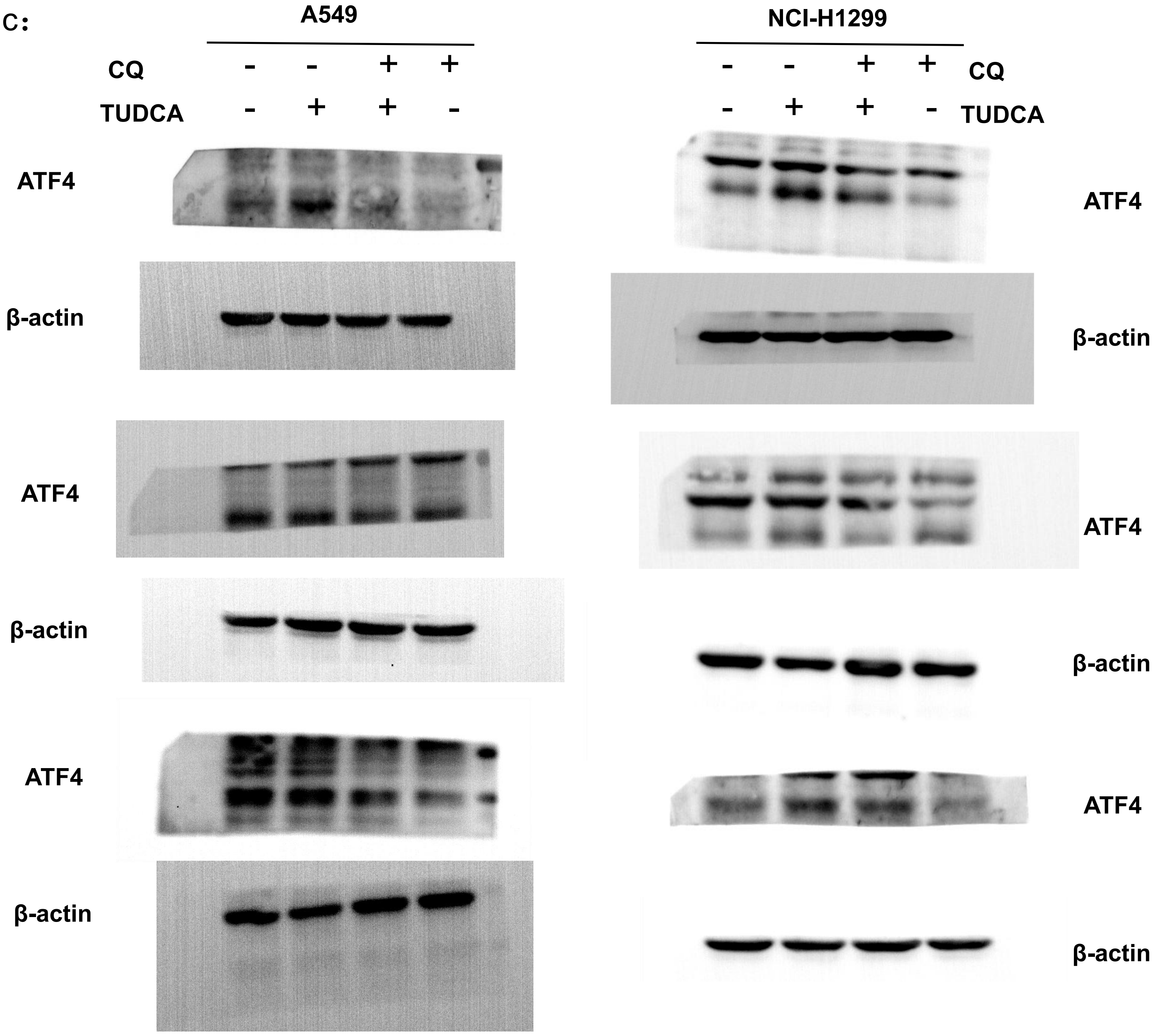
A549
NCI-H1299
CQ - - + +
TUDCA - + + -
ATF4
β-actin
ATF4
β-actin
ATF4
β-actin
- - + + CQ
- + + - TUDCA
ATF4
β-actin
ATF4
β-actin
ATF4
β-actin

Figure 4 c：

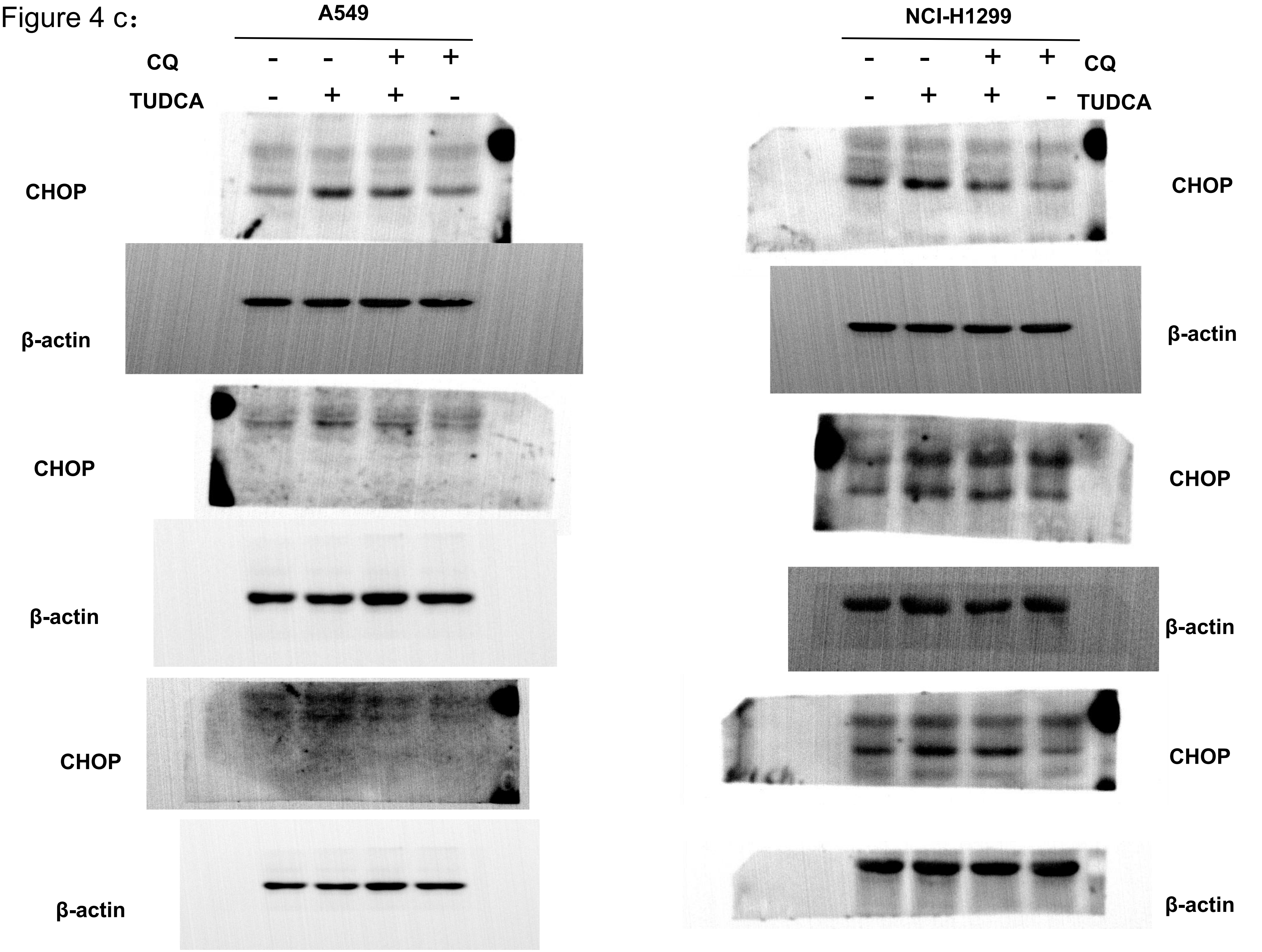
A549
NCI-H1299
CQ - - + +
TUDCA - + + -
CQ - - + +
TUDCA - + + -
CHOP
β-actin
CHOP
β-actin
CHOP
β-actin
CHOP
β-actin
CHOP
β-actin
CHOP
β-actin

Figure 4 c:

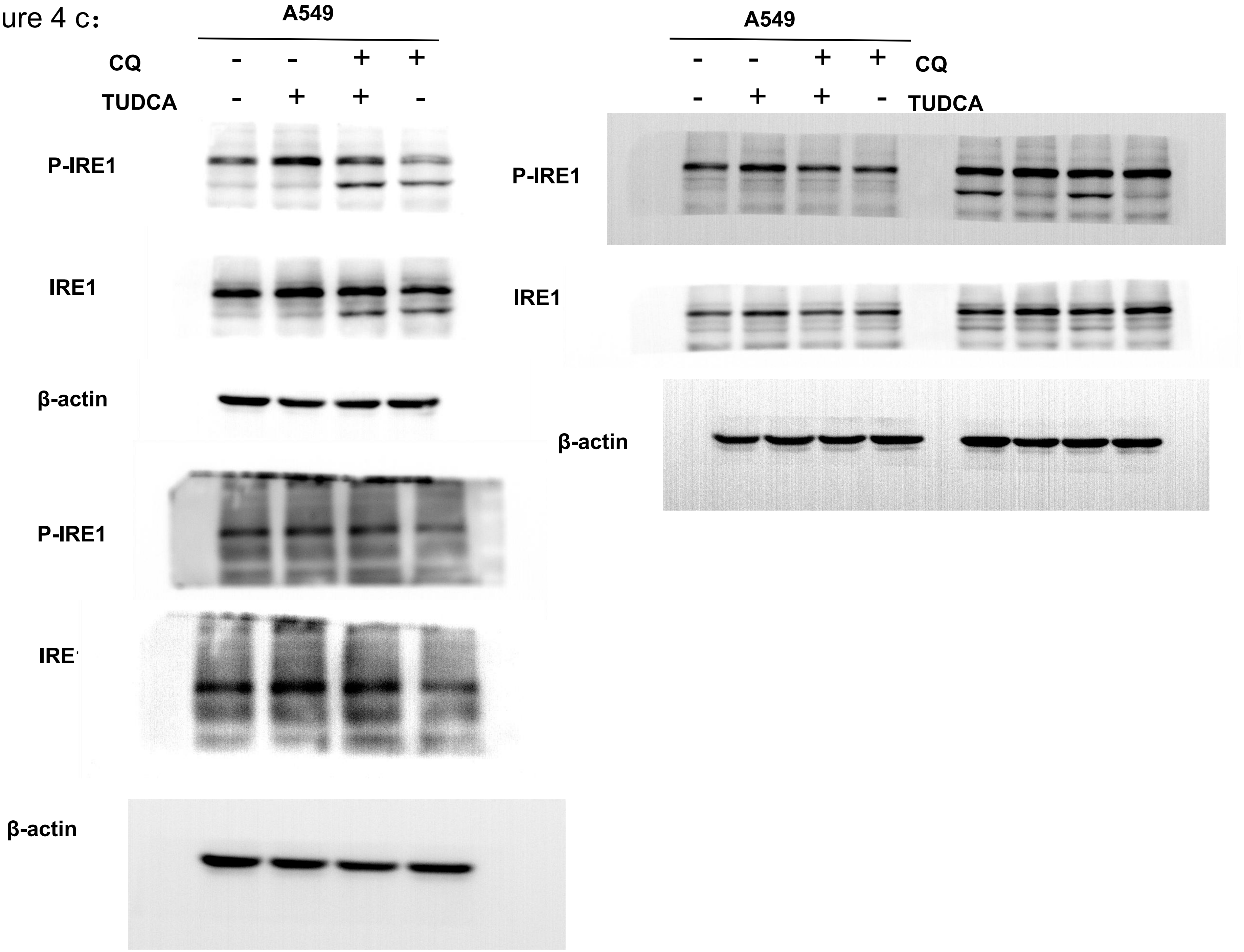
A549
CQ
TUDCA
P-IRE1
IRE1
β-actin
P-IRE1
IRE1
β-actin
A549
CQ
TUDCA
P-IRE1
IRE1
β-actin

Figure 4 c:

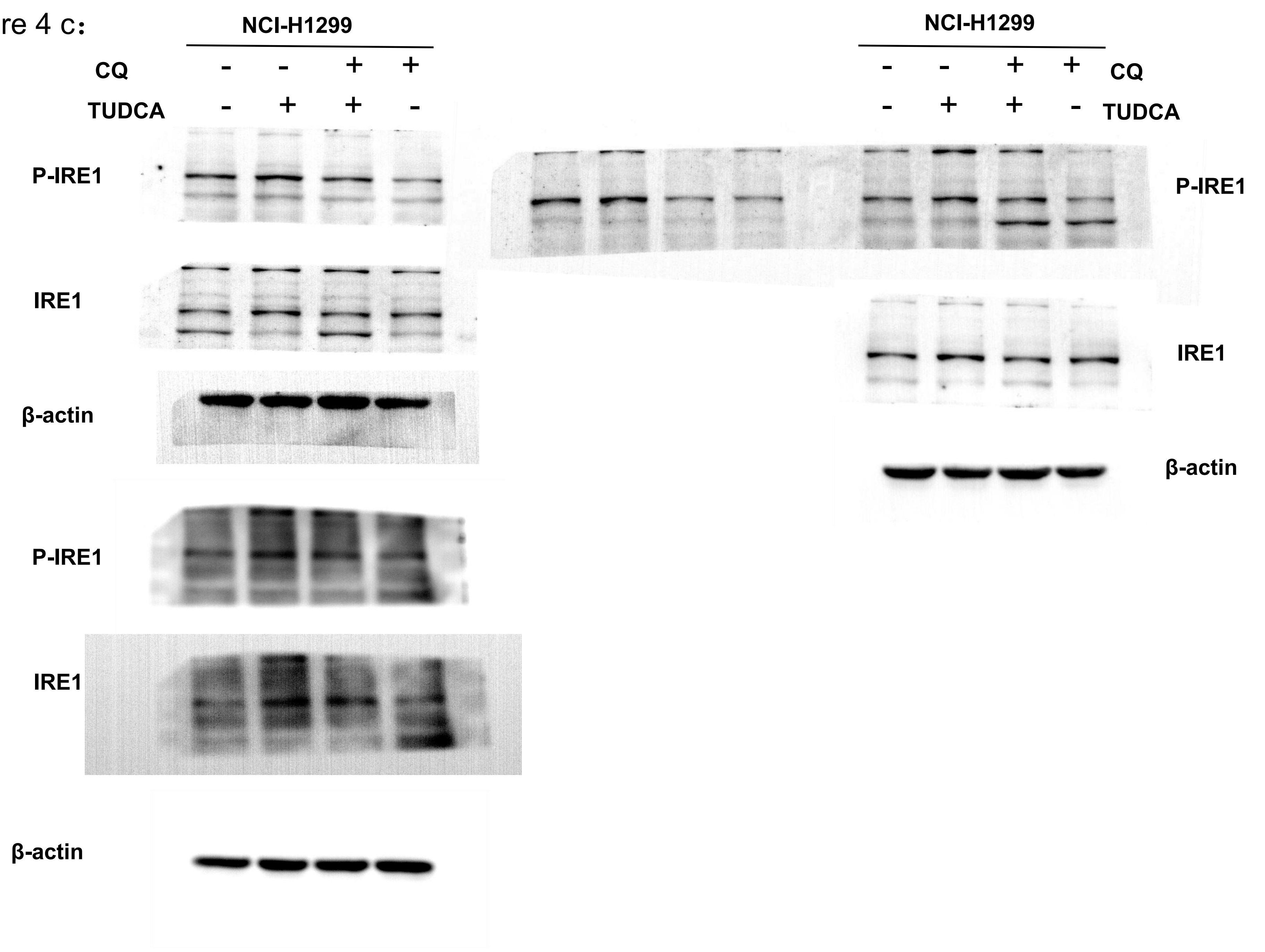
NCI-H1299
CQ - - + +
TUDCA - + + -
P-IRE1
IRE1
β-actin
P-IRE1
IRE1
β-actin
NCI-H1299
- - + + CQ
- + + - TUDCA
P-IRE1
IRE1
β-actin

Figure 4 c:

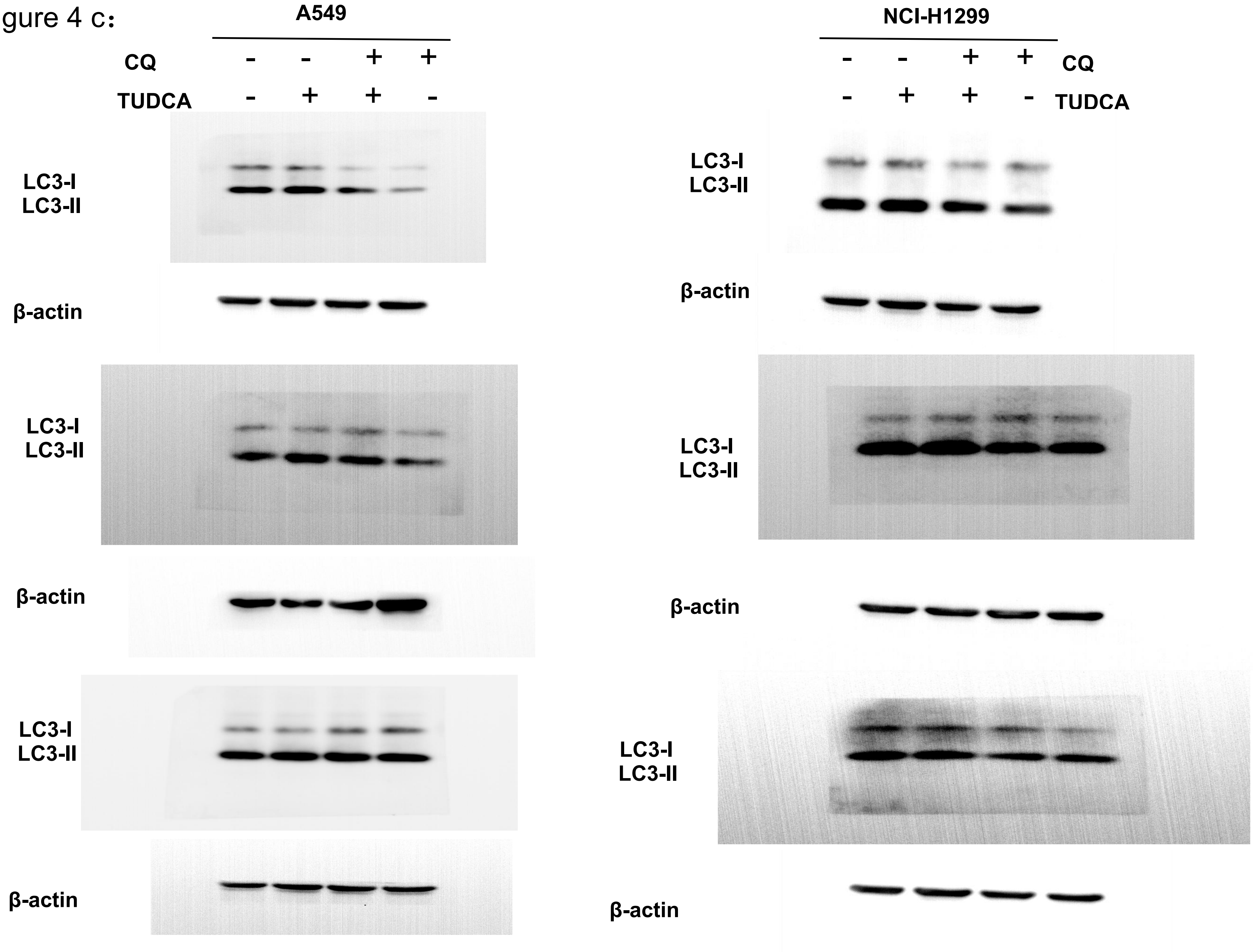
A549
CQ - - + +
TUDCA - + + -
LC3-I
LC3-II
β-actin
LC3-I
LC3-II
β-actin
LC3-I
LC3-II
β-actin
NCI-H1299
- - + + CQ
- + + - TUDCA
LC3-I
LC3-II
β-actin
LC3-I
LC3-II
β-actin
LC3-I
LC3-II
β-actin

Figure 4 c:

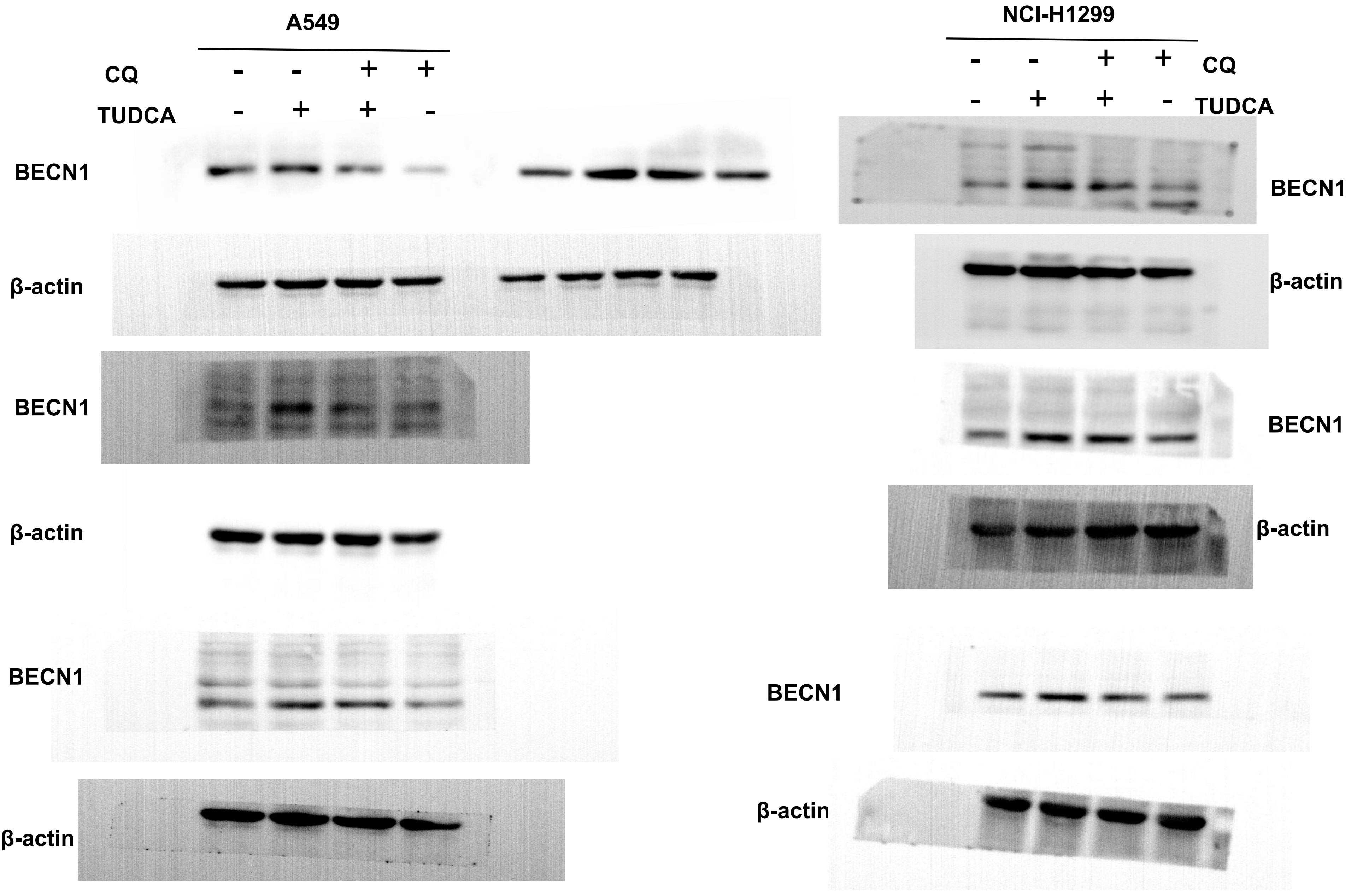
A549
CQ - - + +
TUDCA - + + -
BECN1
β-actin
BECN1
β-actin
BECN1
β-actin
NCI-H1299
- - + + CQ
- + + - TUDCA
BECN1
β-actin
BECN1
β-actin
BECN1
β-actin

Figure 4 c:

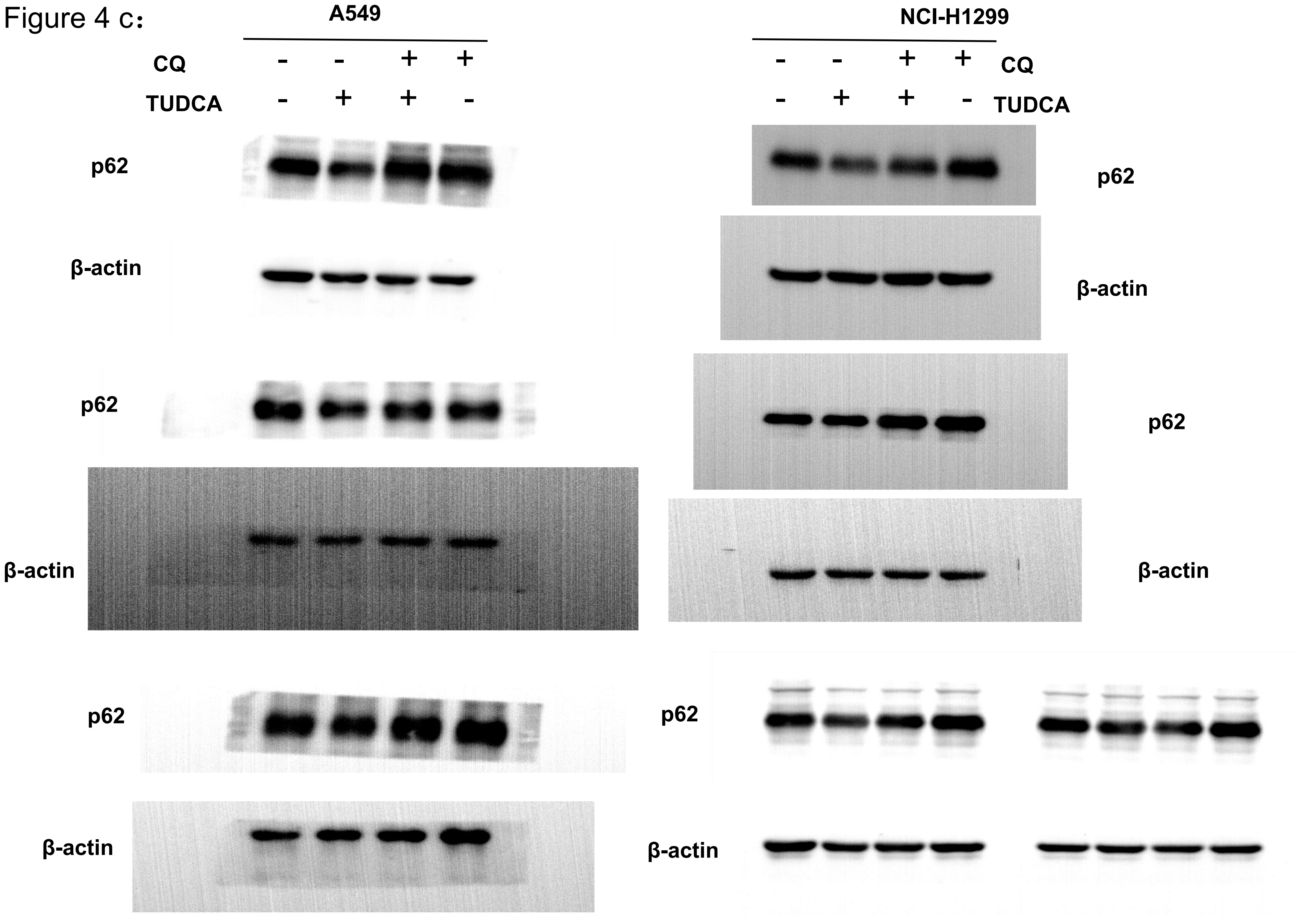
A549
CQ
-
-
+
+
TUDCA
-
+
+
-
p62
β-actin
p62
β-actin
p62
β-actin
NCI-H1299
-
-
+
+
CQ
-
+
+
-
TUDCA
p62
β-actin
p62
β-actin
p62
β-actin

Figure S1：a

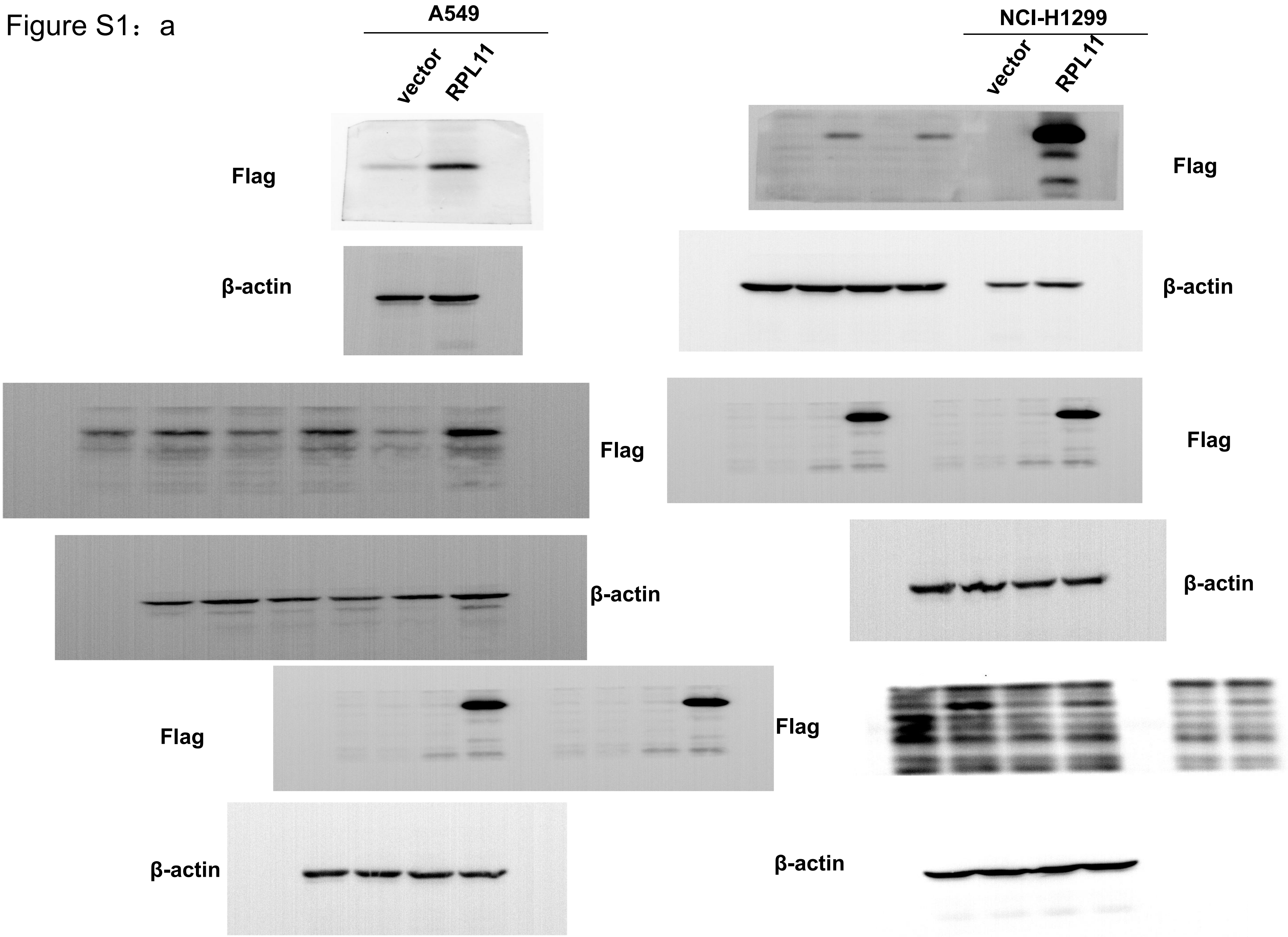
A549
vector
RPL11
NCI-H1299
vector
RPL11
Flag
β-actin
Flag
β-actin
Flag
β-actin
Flag
β-actin
Flag
β-actin
β-actin

Figure S1：c

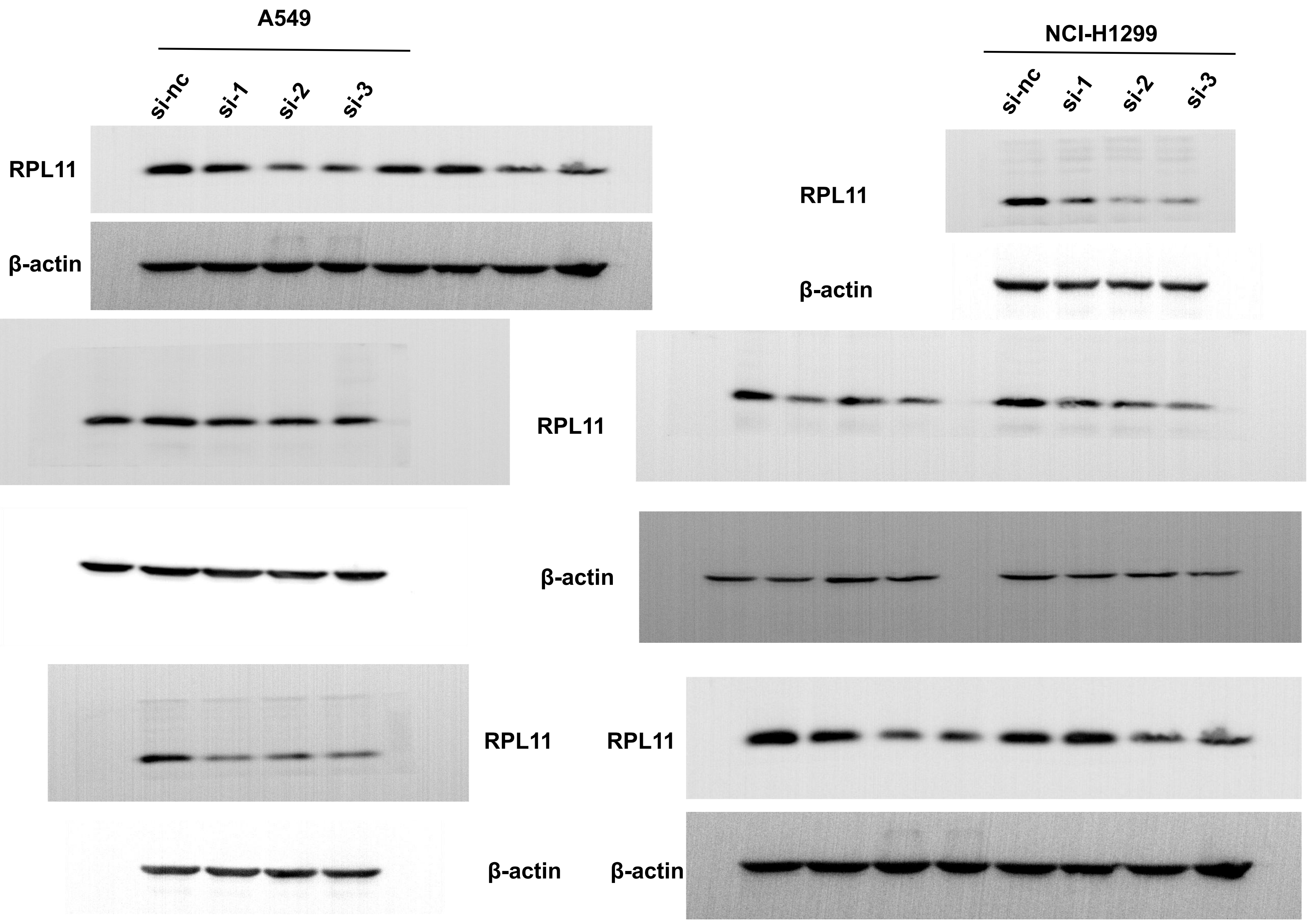
A549
si-nc
si-1
si-2
si-3
RPL11
β-actin
NCI-H1299
si-nc
si-1
si-2
si-3
RPL11
β-actin
RPL11
β-actin
RPL11
β-actin
RPL11
β-actin
RPL11
β-actin
